# *KRAS* mutations in blood circulating cell-free DNA: a pancreatic cancer case-control

**DOI:** 10.18632/oncotarget.12386

**Published:** 2016-10-01

**Authors:** Florence Le Calvez-Kelm, Matthieu Foll, Magdalena B. Wozniak, Tiffany M. Delhomme, Geoffroy Durand, Priscilia Chopard, Maroulio Pertesi, Eleonora Fabianova, Zora Adamcakova, Ivana Holcatova, Lenka Foretova, Vladimir Janout, Maxime P. Vallee, Sabina Rinaldi, Paul Brennan, James D. McKay, Graham B. Byrnes, Ghislaine Scelo

**Affiliations:** ^1^ International Agency for Research on Cancer (IARC), Lyon, France; ^2^ Regional Authority of Public Health, Banska Bystrica, Slovakia; ^3^ Charles University of Prague, First Faculty of Medicine, Institute of Hygiene and Epidemiology, Prague, Czech Republic; ^4^ Masaryk Memorial Cancer Institute and Medical Faculty of Masaryk University, Brno, Czech Republic; ^5^ Department of Preventive Medicine, Faculty of Medicine, Palacky University, Olomouc, Czech Republic; ^6^ Faculty of Medicine, University of Ostrava, Czech Republic

**Keywords:** cell-free DNA, KRAS mutations, plasma, pancreatic cancer detection

## Abstract

The utility of *KRAS* mutations in plasma circulating cell-free DNA (cfDNA) samples as non-invasive biomarkers for the detection of pancreatic cancer has never been evaluated in a large case-control series. We applied a *KRAS* amplicon-based deep sequencing strategy combined with analytical pipeline specifically designed for the detection of low-abundance mutations to screen plasma samples of 437 pancreatic cancer cases, 141 chronic pancreatitis subjects, and 394 healthy controls. We detected mutations in 21.1% (N=92) of cases, of whom 82 (89.1%) carried at least one mutation at hotspot codons 12, 13 or 61, with mutant allelic fractions from 0.08% to 79%. Advanced stages were associated with an increased proportion of detection, with *KRAS* cfDNA mutations detected in 10.3%, 17,5% and 33.3% of cases with local, regional and systemic stages, respectively. We also detected *KRAS* cfDNA mutations in 3.7% (N=14) of healthy controls and in 4.3% (N=6) of subjects with chronic pancreatitis, but at significantly lower allelic fractions than in cases. Combining cfDNA *KRAS* mutations and CA19-9 plasma levels on a limited set of case-control samples did not improve the overall performance of the biomarkers as compared to CA19-9 alone. Whether the limited sensitivity and specificity observed in our series of *KRAS* mutations in plasma cfDNA as biomarkers for pancreatic cancer detection are attributable to methodological limitations or to the biology of cfDNA should be further assessed in large case-control series.

## INTRODUCTION

The latest estimates show that more than 330,000 cases of pancreatic cancer are diagnosed yearly worldwide, and approximately the same number of deaths are attributed to the disease (GLOBOCAN 2012 website: http://globocan.iarc.fr/, accessed on 9 Feb 2015). Disease survival is among the poorest of all cancers with 5-year survival at only 6 % in Europe and ~79 % of patients dying within a year following diagnosis [1, 2]. Improved survival is observed in patients that undergo surgical resection, but this therapeutic option is limited to cases with localized tumors [3]. Early detection has therefore the potential to reduce the mortality associated with pancreatic cancer. Endoscopic ultrasound has shown good sensibility and specificity to detect precancerous and cancerous lesions but this invasive technique has limited use for early detection in asymptomatic individuals [4]. Blood level of the antigen CA 19-9 is the only validated tumor marker for pancreatic cancer with overall sensitivity of 79% (70-90%) and specificity of 82% (68%-91%) [5, 6]. However, non-specific expression in other benign or malignant diseases and absence of expression in Lewis (a-b-) blood phenotypes (~10-15% of the population) limit the use of this biomarker as a diagnostic test [7].

Cell-free DNA fragments (cfDNA) are released into the bloodstream and other body fluids as part of natural cell apoptosis, necrosis and active secretion. Gene mutations in cfDNA fragments have been found to be tumor-specific leading to the concept of circulating tumor DNA (ctDNA) and their potential utility as highly specific non-invasive biomarkers has raised in the recent years [8]. Pancreatic ductal adenocarcinoma (PDAC) accounts for more than 90% of all pancreatic cancer cases [9] and activating hotspot mutations in the *KRAS* gene are present in the majority of them, representing the most frequent [10] but also the earliest genetic alteration that drives pancreatic neoplasia [11–13]. Of the 596 PDAC cases sequenced within the International Cancer Genome Consortium (ICGC) project (https://icgc.org/, as of 23 Feb 2016), 534 (90%) harbored at least one *KRAS* mutation: 83%, 5.5% and 1.5% at codons 12, 61 and 13, respectively. *KRAS* mutations (often restricted to codon 12) have previously been detected in blood (plasma or serum) samples from patients with pancreatic cancer [14–26], showing large variations in the proportion of detected cases (27% to 93%) probably because of inter-laboratory variability, limited sample sizes, and variable sensitivities of the assays. Ultra-deep sequencing technologies allows the identification of low-abundance somatic variants and were shown to be applicable to ctDNA [26–31], but has so far been applied to sample series of limited size and lacking control groups. Here, we investigated whether deep sequencing of *KRAS* codons 12, 13 and 61 in cfDNA from plasma samples from a large series of more than 400 pancreatic cancer cases and 500 controls could represent a comprehensive assay for sensitive and specific detection of pancreatic cancer.

## RESULTS

### Subject characteristics, sequencing performance and inclusion criteria for analysis

Samples were included when cfDNA total yield was at least 4ng and when sequencing reads were above 1000 on average for all codons. In total, 96 samples (100%) from a pilot set and 903 samples (93.4%; 397 pancreatic cancer cases (94.2%); 132 chronic pancreatitis (91.0%) and 374 controls (93.3%)) from a validation set met the inclusion criteria. Table 1 provides the characteristics of cases and controls, as well as the average of cfDNA yields by status. Analysis of variance was used to compare (log-transformed) cfDNA concentrations by subject characteristics listed in table 1 and showed significant difference by status (with higher yields in pancreatic cancer cases versus controls; t-test p<0.0001), stage (higher yields in missing stages versus reported stages: p<0.0001), and center (higher yields in Prague and Olomouc when compared to other centers: p<0.0001). Other variables had no significant influence on cfDNA yield (Fisher test p>0.05).

**Table 1:** Description of the study population

Characteristics	Pilot series (N=96)								Validation series (N=903)					
	Pancreatic cancer cases		Healthy controls		Chronic pancreatitis		Pancreatic benign neoplasms		Pancreatic cancer cases		Healthy controls		Chronic pancreatitis	
	N	%	N	%	N	%	N	%	N	%	N	%	N	%
**Total**	**40**		**20**		**9**		**27**		**397**		**374**		**132**	
**Sex**														
Male	22	*55.0*	11	*55.0*	4	*44.4*	0	*0.0*	220	*55.4*	217	*58.0*	92	*69.7*
Female	18	*45.0*	9	*45.0*	5	*55.6*	0	*0.0*	177	*44.6*	157	*42.0*	40	*30.3*
Missing	0	*0.0*	0	*0.0*	0	*0.0*	27	*100.0*	0	*0.0*	0	*0.0*	0	*0.0*
**Age at blood draw** (mean, sd)	64.8 (10.6)		66.2 (8.7)		62.8 (8.2)		Missing		62.2 (10.2)		60.6 (11.9)		55.6 (12.9)	
**BMI at blood draw** (mean, sd)	24.7 (3.7)		27.4 (4.0)		23.2 (3.8)		Missing		25.1 (4.5)		28.2 (4.3)		24.4 (4.2)	
**Recruiting country**														
Czech Republic	40	*100.0*	20	*100.0*	9	*100.0*	27	*100.0*	298	*75.1*	248	*66.3*	47	*35.6*
Slovakia	0	*0.0*	0	*0.0*	0	*0.0*	0	*0.0*	99	*24.9*	126	*33.7*	85	*64.4*
**Tobacco smoking**														
Never	20	*50.0*	9	*45.0*	3	*33.3*	0	*0.0*	167	*42.1*	175	*46.8*	45	*34.1*
Ex-smoker	10	*25.0*	6	*30.0*	4	*44.4*	0	*0.0*	123	*31.0*	113	*30.2*	24	*18.2*
Current smoker	10	*25.0*	5	*25.0*	2	*22.2*	0	*0.0*	107	*27.0*	86	*23.0*	63	*47.7*
Missing	0	*0.0*	0	*0.0*	0	*0.0*	27	*100.0*	0	*0.0*	0	*0.0*	0	*0.0*
**Alcohol drinking**														
Never	25	*62.5*	12	*60.0*	4	*44.4*	0	*0.0*	212	*53.4*	176	*47.1*	49	*37.1*
Ex-drinker	6	*15.0*	3	*15.0*	4	*44.4*	0	*0.0*	95	*23.9*	36	*9.6*	48	*36.4*
Current drinker	9	*22.5*	5	*25.0*	1	*11.1*	0	*0.0*	87	*21.9*	162	*43.3*	35	*26.5*
Missing	0	*0.0*	0	*0.0*	0	*0.0*	27	*100.0*	3	*0.8*	0	*0.0*	0	*0.0*
**Tumor stage at diagnosis**														
Local	6	*15.0*	-		-		-		33	*8.3*	-		-	
Regional	17	*42.5*	-		-		-		126	*31.7*	-		-	
Systemic	16	*40.0*	-		-		-		119	*30.0*	-		-	
Unknown	1	*2.5*	-		-		-		119	*30.0*	-		-	
**Tumor histological type**														
Ductal adenocarcinoma	40	*100.0*	-		-		-		243	*61.2*	-		-	
Other ductal carcinoma	0	*0.0*	-		-		-		19	*4.8*	-		-	
Endocrine	0	*0.0*	-		-		-		14	*3.5*	-		-	
Missing/Unknown	0	*0.0*	-		-		-		121	*30.5*	-		-	
**Log10 cfDNA concentration, ng/mL plasma** (mean, sd)	1.7 (0.5)		1.7 (0.5)		1.8 (0.3)		1.9 (0.7)		2.0 (0.7)		1.7 (0.6)		1.8 (0.7)	

The average mean depth of reads after filtering on mapping quality were, for the pilot and validation sets, respectively: 3992 (SD= 1123) and 2888 (SD=1259) at *KRAS* codon 12 c.34, and 2492 (SD=710) and 3765 (SD=1762) at codon 61 c.181.

### Determination of the allelic fraction threshold for the detection of the *KRAS* p.G12V variant

**Figure 1:**
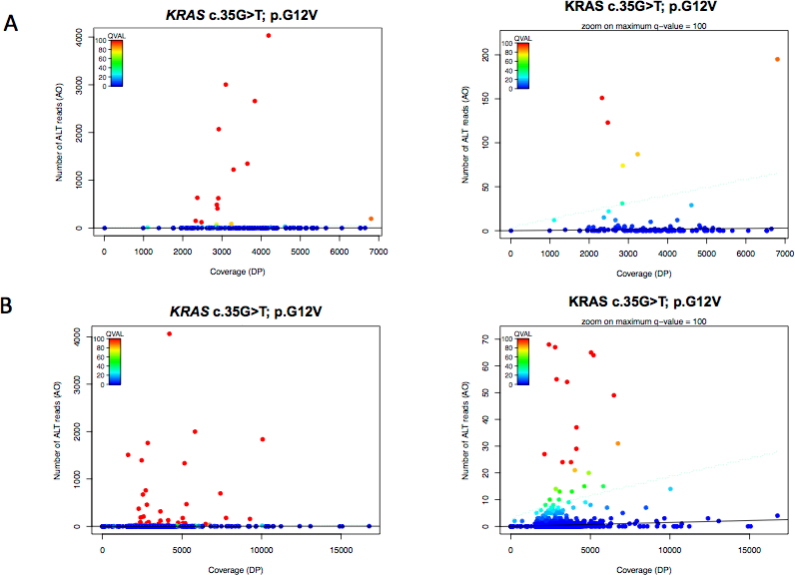
Mutation detection of *KRAS* c.35G>T; p.G12V in serial dilution and cfDNA samples using the Needlestack approach Negative-binomial regression plot at *KRAS* c.35G>T; p.G12V displaying the total number of reads (coverage, DP) and the number of reads matching the candidate variant (AO). Black solid line: Estimated error rate (e) at the c.35 position for this G>T base change. Blue dashed line: Detection limit at q-values <10^−3^; >30 in Phred scale (QVAL). Dots above the blue dashed line: Outliers of the regression (QVAL≥30), declared as mutant *KRAS* samples (c.35G>T; p.G12V). Dots below the blue dashed line: Inliers (QVAL<30) declared unmutated at this position for specified base change. **A.** Serial dilution of SW480 cell-lines in duplicates (N=28) and cfDNA from the pilot series (N=96) sequenced on a Ion Torrent PGM 316 Chip (e= 4.2×10^−4^); **B.** Serial dilution of SW480 cell-lines in duplicates (N=28) and cfDNA from the validation series (N=903) sequenced on Ion Torrent PGM 318 chips (e=1.4×10^−4^).

The number of reads obtained from sequencing of 2ng of two independent serial dilutions (in duplicates) of *KRAS* c.35G>T; p.G12V mutated DNA was between 991 and 4205 with an average read depth of 2693 (Supplementary Table S1, Supplementary Data). There was a good correlation between expected and observed mutant allelic fractions (r2=0.948; Supplementary Figure S1). Needlestack analysis was performed independently on the 2 sets of data (Figure 1). Phred scale q-values (QVAL) determined by the Negative binomial regression show that the *KRAS* p.G12V mutation could be reliably detected down to a minor allele frequency of 0.2% when read depth was approximately of 2500 reads QVAL>30 for 3 of the replicates at 0.2%) (Supplementary Table S1, Supplementary Data).

### Performance of *KRAS* mutations in cfDNA samples in complement to CA19-9 plasma levels as non-invasive pancreatic cancer biomarker

Applying a threshold of QVAL >30 to the sample set of the pilot series, *KRAS* mutations at hotspot codons reported in PDAC were identified in cfDNA plasma samples in 7 of 40 cases (sensitivity 17.5%) with PDAC and in 1 cfDNA of 27 patients with pancreatic benign neoplasms. None were detected in healthy controls, or in patients with chronic pancreatitis (overall specificity of 98.2%; of 100% against healthy controls) (Tables 2 and 3). All *KRAS* mutations were located at codon 12 (See Supplementary Data Supplementary Table S2 for the complete list of samples harboring cf DNA *KRAS* mutations). Investigating the presence of *KRAS* mutations at other screened codons (from *KRAS* codons 4 to 16 and from codons 51 to 69) and reported mutated for any cancer sites in the COSMIC database identified (i) 2 additional PDAC cases with cfDNA *KRAS* mutations (1 case with p.K5R and 1 case with p.K5R and p.G10R; leading to an overall sensitivity of 22.5%) and (ii) 1 additional mutation in a patient with benign neoplasm of the pancreas (p.A11P). All mutations except one had allelic fraction below 3% (Supplementary Table S2, Supplementary Data).

**Table 2:** *KRAS* mutations and CA19-9 plasma levels in the pilot series (N=94)

	cfDNA *KRAS* mutation at hotspot codons (12, 13, 61) reported in PDAC		cfDNA *KRAS* mutation at any screened codons reported in any cancer sites	
	N	%	N	%
**Plasma CA19-9 positive level (≥37Ku/l)**				
PDAC case, N=36	5	*13.9*	7	*19.4*
Healthy controls, N=3	0	*0.0*	0	*0.0*
Benign pancreatic neoplasm, N=11	1	*9.1*	1	*9.1*
Chronic pancreatitis, N=5	0	*0.0*	0	*0.0*
**Plasma CA19-9 negative level (<37Ku/l)**				
PDAC case, N=4	2	*50.0*	2	*50.0*
Healthy controls, N=17	0	*0.0*	0	*0.0*
Benign pancreatic neoplasm, N=14	0	*0.0*	1	*7.1*
Chronic pancreatitis, N=4	0	*0.0*	0	*0.0*
**Total**				
PDAC case, N=40	7	*17.5*	9	*22.5*
Healthy controls, N=20	0	*0.0*	0	*0.0*
Benign pancreatic neoplasm, N=25*	1	*4.0*	2	*8.0*
Chronic pancreatitis, N=9	0	*0.0*	0	*0.0*

* Two benign neoplasms were excluded from this analysis because CA19-9 plasma level measurements could not be performed.

**Table 3:** Performance of NGS-based assay for the detection of cfDNA *KRAS* mutations, CA19-9 plasma level and combined assays (40 PDAC, 20 healthy controls, 9 chronic pancreatitis subjects, and 25 benign neoplasm subjects)

	Sensitivity	Overall Specificity*	Specificity against healthy controls
**cfDNA *KRAS* mutation**			
at PDAC hotspot codons (12, 13, 61)	17.5%	98.2%	100.0%
at any screened codons reported in any cancer sites	22.5%	96.4%	100.0%
**CA19-9 plasma level (≥37Ku/l)**	90.0%	64.8%	85.0%
**Combined cfDNA *KRAS* mutation and CA19-9 plasma level**			
at PDAC hotspot codons (12, 13, 61)	95.0%	64.8%	85.0%
at any screened codons reported in any cancer sites	95.0%	63.0%^a^	85.0%

* against non-PDAC and controls

a Decreased specificity due to the detection of c.31G>C; p.A11P *KRAS* mutation in a patient with benign neoplasm negative for the plasma CA19-9 assay

The sensitivity and the overall specificity of plasma CA19-9 levels for detecting PDAC was 90.0% and 64.8% respectively (Table 3). Combining these so that the test was declared positive if a *KRAS* mutation was found at any COSMIC reported position or if the CA19-9 plasma level was positive enabled the detection of 2 additional PDAC cases (38/40) that were negative for CA19-9 plasma level but positive for cfDNA *KRAS* mutation, increasing the sensitivity to 95% (Tables 2 and 3). Comparisons of AUCs of the combined assays versus CA19-9 levels alone showed small increases, approximately 0.02 for each of the three comparisons (cancer cases vs. healthy controls; cancer cases vs. all other conditions; cancer cases vs. benign pancreatic conditions) and were non significant (p>0.17 for all comparisons).

### Validation of the proportions of detectable cfDNA *KRAS* mutations in pancreatic cancer cases and controls

We extended the cfDNA *KRAS* mutation screening to the validation case-control series (N=903) (Table 1). Of the 397 patients with pancreatic cancer, 75 (18.9%) carried at least one cfDNA *KRAS* mutation at PDAC hotspot codons, a sensitivity close to that reported for the pilot series (17.5%). We also detected at least one *KRAS* mutations at PDAC hotspot codons in the plasma of 4/132 (3.0%) patients with chronic pancreatitis and of 9/374 (2.4%) healthy controls whereas none were detected in those subjects of the pilot series. Enlarging the search for *KRAS* mutations to other screened codons increased the sensitivity to 20.9% (83 patients with pancreatic cancer carrying at least one mutations in their cfDNA), but decreased the specificity with the detection of cfDNA *KRAS* mutations in 6/132 (4.5%) patients with chronic pancreatitis and in 14/374 (3.7%) healthy controls (Table 4).

**Table 4:** Proportion of subjects with *KRAS* mutations in their plasma cfDNA

	Pancreatic cancer cases						Chronic pancreatitis						Healthy controls					
	All N=437		Pilot N=40		Validation N=397		All N=141		Pilot N=9		Validation N=132		All N=394		Pilot N=20		Validation N=374	
	N	%	N	%	N	%	N	%	N	%	N	%	N	%	N	%	N	%
**Subjects with *KRAS* mutations in cell-free DNA**	**92**	***21.1***	**9**	**22.5**	**83**	***20.9***	**6**	***4.3***	**0**	***0.0***	**6**	***4.5***	**14**	***3.6***	**0**	***0.0***	**14**	***3.7***
***Numbers of mutation***																		
Single	89	*20.4*	8	20.0	81	*20.4*	4	*2.8*	0	*0.0*	4	*3.0*	11	*2.8*	0	*0.0*	11	*2.9*
Multiple	3	*0.7*	1	2.5	2	*0.5*	2	*1.4*	0	*0.0*	2	*1.5*	3	*0.8*	0	*0.0*	3	*0.8*
***Location***																		
Mutation(s) at PDAC hotpot codon(s) 12, 13 or 61	81	*18.5*	7	17.5	74	*18.6*	4	*2.8*	0	*0.0*	4	*3.0*	8	*2.0*	0	*0.0*	8	*2.1*
Mutation(s) at other codon(s)*	10	*2.3*	2	5.0	8	*2.0*	2	*1.4*	0	*0.0*	2	*1.5*	5	*1.3*	0	*0.0*	5	*1.3*
Mutations at hotpot codons 12, 13 or 61 and others*	1	*0.2*	0	0.0	1	*0.3*	0	*0.0*	0	*0.0*	0	*0.0*	1	*0.3*	0	*0.0*	1	*0.3*
***Type***																		
Missense	92	*21.1*	9	22.5	83	*20.9*	5	*3.5*	0	*0.0*	5	*3.8*	12	*3.0*	0	*0.0*	12	*3.2*
Silent	0	*0.0*	0	0.0	0	*0.0*	1	*0.7*	0	*0.0*	1	*0.8*	2	*0.5*	0	*0.0*	2	*0.5*

* Codons reported mutated in COSMIC (all cancer sites)

We identified 3 silent base substitutions c.24A>G p.V8V (2 in cases and one in controls), which we considered as a rare SNP (rs147406419) as it was reported with an allelic frequency between 0.0002 (Exome Variant Server ESP6500siv2) and 0.0004 (Exome Aggregation Concortium ExAC) and classified as probably non-pathogenic impact by CLINSIG (Table S3, Supplementary Data). The 3 base substitutions are consequently not included in this table.

Of note, we identified 3 subjects (2 cases and 1 control) with the silent base substitution c.24A>G p.V8V (at 46.38%, 11.46% and 46.98% allelic fractions respectively) which we considered as a rare SNP (rs147406419) as it was reported with an allelic frequency between 0.02% (Exome Variant Server ESP6500siv2) and 0.04% (Exome Aggregation Concortium ExAC) and classified as probably non-pathogenic impact by CLINSIG. This variant was ignored for the rest of the analysis. Further restricting the analysis to missense *KRAS* mutations decreased false positive rates to 3.8% (5/132) and 3.2% (12/374) respectively (Table 4). The complete list of *KRAS* mutations identified in cfDNA of the validation series and corresponding allelic fractions is available in supplementary data (Supplementary Data, Supplementary Table S3). The lowest allelic fraction detected in the cfDNA samples was 0.08% in a plasma case (sample CA93) at *KRAS* p.G13R (Supplementary data, Supplementary Figure S2).

**Figure 2:**
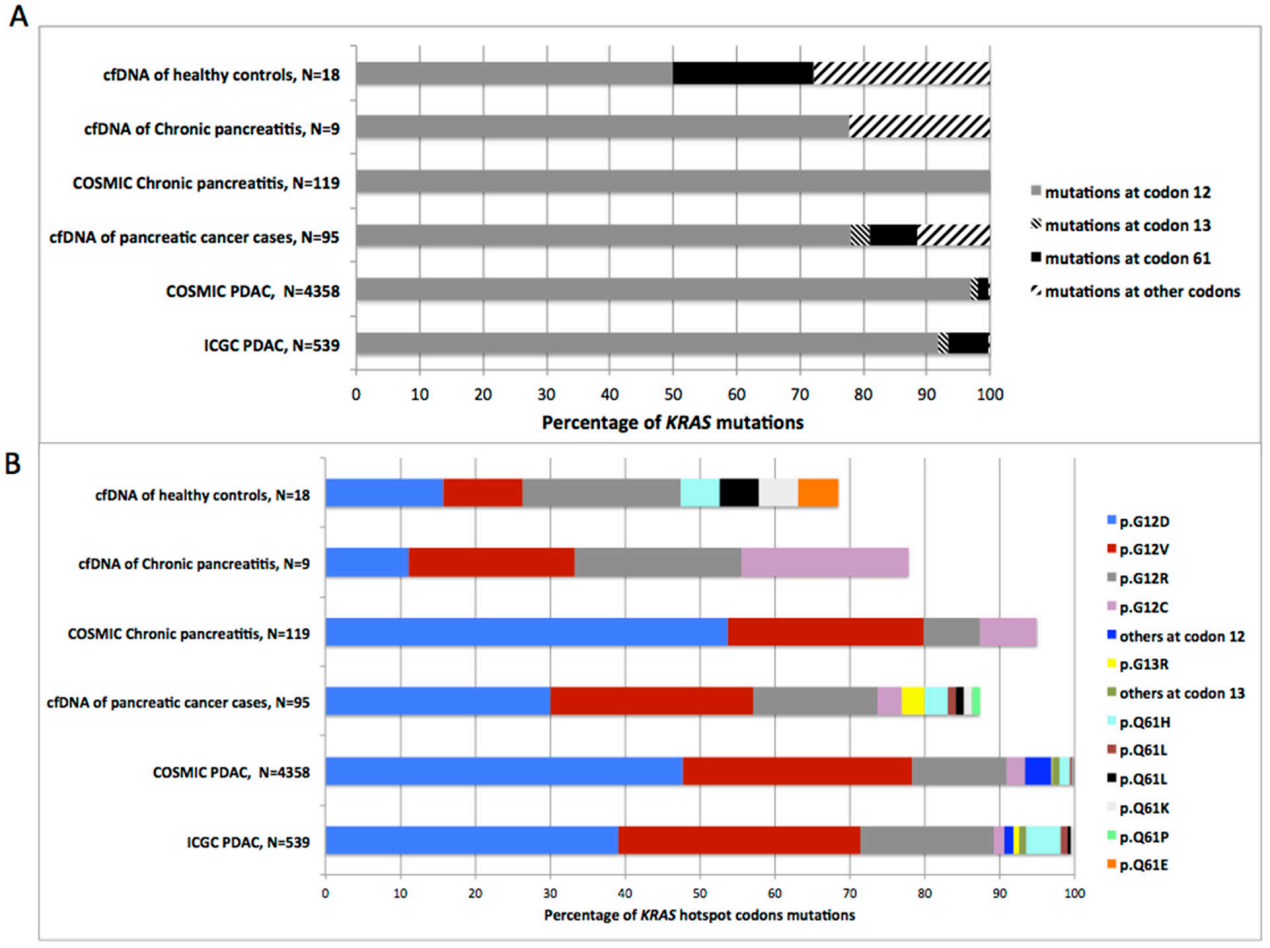
Distribution of *KRAS* mutations detected in plasma samples from pancreatic cases, chronic pancreatitis and healthy controls compared to somatic *KRAS* mutations reported in ICGC and COSMIC database **A.** Comparison of cfDNA *KRAS* mutation location; **B.** Comparison of *KRAS* mutation spectrum at hotspot codons (12, 13 and 61). N= Number of *KRAS* mutations.

As for somatic *KRAS* mutations reported in PDAC (COSMIC and ICGC data) and chronic pancreatitis (COSMIC data), the majority of cfDNA *KRAS* mutations identified in the combined pilot and validation series were located at codon 12 (76.3 % in pancreatic cancer cases; 77.8% in chronic pancreatitis and 47.4% in healthy controls; Figure 2A). Similar proportions of *KRAS* mutations at codons 61 and 13 were observed in cfDNA of pancreatic cancer cases (7.2% and 3.1% respectively) as compared to PDAC ICGC tumors (6.1% and 1.7% respectively). However, while less than 1% of *KRAS* mutations reported in ICGC/COSMIC data are located at other codons, 13% (13/97), 22% (2/9), and 31% (6/19) of such mutations were detected in the plasma samples of cancer cases, chronic pancreatitis, and controls, respectively (Figure 2A). The frequencies of the most predominant mutation types reported for PDAC in ICGC, i.e p.G12D, p.G12V, p.G12R, p.G12C followed by p.Q61H, p.Q61R and p.Q61L paralleled the frequencies of the cfDNA *KRAS* mutations in cases (Figure 2B) reflecting the probable tumor origin of the cfDNA *KRAS* mutations. In addition, one cancer case and one control harbored p.Q61P and p.Q61E in their cfDNA, respectively, two non-PDAC COSMIC missense substitutions previously reported in various cancer tissues (Figure 2 and Supplementary Data Supplementary Table S4).

We did not observe striking differences by histological groups. Amongst the 283 PDAC cases, 59 (20.8%) were detected with a cfDNA *KRAS* mutation, all but three (p.M67L, p.M72V, and p.Q61P) reported as predominant PDAC mutations. Four “other ductal carcinoma” cases out of 19 (21.1%) were also detected with a single cfDNA mutation, all four reported as hotspot PDAC mutations. Amongst the 16 endocrine cases, 3 mutations were detected in 3 cases (18.7%), two of them not reported as hotspot PDAC mutations (p.G60D and p.A59G). The two cases with multiple mutations were found in the pancreatic cancer cases of unknown histological type, where 29 mutations were detected in 27/121 (22.3%) cases. Of these 29 mutations, five (p.A59E, p.E62D, p.Q61R, p.Q70P, and p.Y64D) were not reported as predominant PDAC mutations.

Advanced stages were significantly associated with an increased proportion of detection (*KRAS* cfDNA mutations were detected in 10.3% of cases diagnosed with local stage, 17,5% with regional stage, and 33.3% with systemic stage; chi-squared p=0.0009) (Table 5). Among detected cases, there was a non-significant trend of increased allelic fractions with stage (log10 of fractions were 0.1270, 0.1349, and 0.3047) on average, for local, regional and systemic disease, respectively; linear regression t-test p=0.3278). Allelic fractions correlated significantly with status (Table 6), pancreatic cancer cases carrying cfDNA *KRAS* mutations at higher allelic fractions than patients with chronic pancreatitis (t-test on log10(allelic fractions) p=0.0259) and healthy controls (p=0.0008). Healthy controls and chronic pancreatitis subjects had similar allelic fractions (p=0.8218). Of note, 3 PDAC cases were found to carry *KRAS* mutations in their plasma samples at allelic fractions higher than 50% reflecting gain of mutant *KRAS* copies. Other factors associated with allelic fractions were: histological type (with “other ductal carcinoma” cases having higher allelic fractions than PDAC (p=0.0016), endocrine (p=0.0078), and unknown/missing types (p=0.0004); sex (males having higher allelic fractions than females in healthy controls, p=0.0069); and age (borderline trend showing higher allelic fractions in older controls, p=0.0548).

**Table 5:** Proportion of pancreatic cancer cases with *KRAS* mutations in their plasma cfDNA, by stage

Stage	Pilot series			Validation series			All		
	Total	cfDNA *KRAS* mutation		Total	cfDNA *KRAS* mutation		Total	cfDNA *KRAS* mutation	
	N	N	%	N	N	%	N	N	%
Local	6	1	*16.7*	33	3	9.1	39	4	*10.3*
Regional	17	1	*5.9*	126	24	19.0	143	25	*17.5*
Systemic	16	7	*43.8*	119	38	31.9	135	45	*33.3*
Unknown	1	0	*0.0*	119	18	15.1	120	18	*15.0*
**All**	**40**	**9**	***22.5***	**397**	**83**	**20.9**	**437**	**92**	***21.1***

*KRAS* Mutations identified at hotspot codons 12, 13 and 61 and at other codons reported mutated in COSMIC; the silent base substitution c.24A>G p.V8V was excluded from analysis.

**Table 6:** Proportion of subjects with cfDNA *KRAS* mutations at various allelic fractions

^a^AF (%)	Pancreatic cancer cases						Chronic pancreatitis						Healthy controls					
	All, N=93		Pilot, N=9		Validation, N=84		All, N=6		Pilot, N=0		Validation, N=6		All, N=14		Pilot, N=0		Validation, N=14	
	N	%	N	%	N	%	N	%	N	%	N	%	N	%	N	%	N	%
<0.2	4	*4.3*	0	*0.0*	4	*4.8*	1	*16.7*	0	*0.0*	1	*16.7*	4	*28.6*	0	*0.0*	4	*28.6*
[0.2-1]	35	*37.6*	3	*33.3*	32	*38.1*	4	*66.7*	0	*0.0*	4	*66.7*	7	*50.0*	0	*0.0*	7	*50.0*
[1.01-10]	40	*43.0*	5	*55.6*	35	*41.7*	0	*0.0*	0	*0.0*	0	*0.0*	2	*14.3*	0	*0.0*	2	*14.3*
[10.01-50]	11	*11.8*	1	*11.1*	10	*11.9*	1	*16.7*	0	*0.0*	1	*16.7*	1	*7.1*	0	*0.0*	1	*7.1*
[50.01-79]	3	*3.2*	0	*0.0*	3	*3.6*	0	*0.0*	0	*0.0*	0	*0.0*	0	*0.0*	0	*0.0*	0	*0.0*

a AF : Allelic Fraction

*KRAS* p.V8V excluded

For samples with multiple variants, the mutation with the highest allelic frequency was taken into account

## DISCUSSION

To our knowledge, our study is the largest screening of *KRAS* mutations in plasma samples of pancreatic cancer cases, other pathological pancreatic conditions and healthy controls allowing for the comprehensive assessment of the sensitivity and specificity of *KRAS* mutations as non-invasive biomarkers for the detection of pancreatic cancer. Using only 2ng/amplicon (4ng total) of cfDNA and amplicon sizes below the size of the most prominent peak (166 bp) of the recently reported narrow range distribution of cfDNA fragments size [32], our NGS-based *KRAS* mutation screening assay combined with our developed Needlestack variant caller algorithm proved to be a sensitive approach to detect low-allelic fraction *KRAS* mutations down to 0.08%; a detection limit comparable to other amplicon-based NGS sequencing methods [27, 30, 31, 33].

We demonstrated that cfDNA *KRAS* mutations were detectable at the time of diagnosis in the plasma of 20% of pancreatic cancer cases at PDAC hotspot codons (12, 13 and 61); a sensitivity which is more consistent with some studies (between 27 to 36%) [14, 16, 20, 25] than others (between 47 to 81%) [15, 17–19]. As previously reported, the majority of these alterations were located at the hotspot codon 12, the spectrum was concordant with the distribution of *KRAS* tumor mutation types from ICGC data [34–36], suggesting that *KRAS* mutations in the circulating DNA mainly originate from tumor cells. Interestingly, although it has been shown that 90% of patients with PDAC carry primary *KRAS* mutations at codons 12, 13 or 61, we identified 9 cfDNA variants outside of the predominantly mutated codons, not reported in the ICGC PDAC database but reported in the COSMIC database for other types of cancer, allowing for an increased sensitivity of 22.5%. Those non-hotspot cfDNA *KRAS* mutations identified in pancreatic cancer cases may reflect the heterogeneity of the tumors or the alterations of genetically different metastatic lesions. In agreement with previous reports, we also demonstrated that the proportion of cases with detectable cfDNA *KRAS* mutations tended to increase with more advanced stages and that *KRAS* allelic fractions were higher in cases than controls or in patients with chronic pancreatitis [23, 26]. Using a ddPCR assay focusing on the four most common PDAC mutations (G12D, G12V, G12R, G13D) Takai and colleagues identified cfDNA *KRAS* mutations in PDAC patients with distant organ metastasis in higher proportion than us (58.9% and 33.3% respectively). However, both studies report similar proportion of detected cases in non-metastatic and localized disease; 8.3% of patients with resectable PDAC (stages I and II) in Takai study and 10.3% of patients with localized pancreatic cancer in our study [23]. While a recent study using ddPCR demonstrated a higher sensitivity (43%; 22 patients) for the detection of *KRAS* mutation in plasma samples of patients with localized PDAC, 10 patients harbored a mutation at an allelic fraction ≤ 0.08% [22]. As 0.08% represents the lowest allele fraction that we could detect with our NGS-based approach and Needlestack algorithm, it is likely that some true low-allelic fraction mutants were too close to the sequencing noise signals to be detected at QVAL> 30. A combined strategy of pre-screening by NGS-amplicon followed by ddPCR of suggestive but inconclusive samples for specific mutations (for example samples with 10<QVAL<30) could circumvent some limitations by discriminating true positive low-level allele fractions mutants from inconclusive or false negative NGS samples, providing that the amount of cfDNA obtained is not a limiting factor. Preanalytical parameters regarding blood processing are also known to affect cfDNA concentrations [37]. A limitation of our study is that we did not test whether removing cellular debris with a high speed centrifugation of plasma samples prior cfDNA isolation could improve the sensitivity. However, the low quantities of cfDNA we could extract from the plasma samples on average indicate that contamination by cellular DNA was minimal. It is possible that a proportion of KRAS mutant pancreatic cancer do not release KRAS mutant cfDNA in the bloodstream, in which case the main limiting factor would be the biology of the tumor rather than the technology. Whether those differences in the release process of ctDNA between patients are due to differences in tumor micro-environment, vascularization, molecular characteristics and/or clonality remains to be discovered [38, 39].

Our study highlights that at our level of detection, a non-negligible proportion of controls are detected. Sausen and colleagues report 99.9% specificity of their assay against matched tumor DNA but they have not evaluated the specificity of their method against plasma of healthy controls. This becomes of capital importance when ultra-low detection limit is required as the proportion of positive calls in non-cancer individuals is likely to increase significantly. The assessment of the biological specificity of mutations in cfDNA as a non-invasive biomarker is either inexistent or limited in size. This may be partly explained by the fact that somatic mutations are believed to occur at negligible frequencies in normal cell populations [40], and thus expected to derive exclusively from the tumor burden. Yet, using a technique of limited sensitivity, Gormally et al. reported the presence of *KRAS* (1%) and *TP53* (3.2%) mutations in plasma of individuals who had remained clinically cancer-free for more than five years [41]. Very recently two studies revealed low-abundant *TP53* somatic mutations in body fluids of non-cancer individuals [42,43]. In addition, while limited in sample size, two studies described circulating *KRAS* mutations in 5% (2/37) [14] and 13% (4/31) [17] of patients with chronic pancreatitis. In our series, we detected 3.7% (N=14) *KRAS* positive individuals in the healthy controls (N=9 at hotspot codons) and 4.3% (N=6) in subjects with chronic pancreatitis, three of them at PDAC hotspot codon with an allelic fraction >1%. Given the prevalence of *KRAS* mutated cancers (predominantly pancreas, colon and lung) in the population, we cannot exclude that a small proportion of these individuals were non-diagnosed *KRAS* mutated cancer cases. Cell-free DNA fragments released into the blood circulation represent a molecular footprint of the entire genome, potentially including somatic mutations that occur at a mosaic state e.g affecting a limited number of tissues and cells. Syndromes caused by mosaic mutations in the Ras/MAPK signaling pathway (Mosaic RASopathies) have been described as a rather frequent congenital disorder that results in special skin phenotypes, whose epidermal and sebaceous disorders have been recently attributed, among other mutations, to oncogenic mosaic *KRAS* mutations [44]. The relatively high incidence of the most frequent mosaic RASopathy; sebaceous nevi (1 in 1,000 births) suggest that *KRAS* mutations present at a mosaic state in humans may not be a rare phenomenon [45]. Moreover, mosaic RASopathies are predominantly reported as skin disorders because of the accessibility of the lesions but the frequency of those syndromes could be underestimated as mosaic RASopathies of internal organs have been poorly investigated. While there are no accurate estimates of the prevalence and pathogenicity of mosaic *KRAS* mutations in human, it is possible that a proportion of cancer-free individuals with detectable low allelic fractions mutations in circulating DNA could reflect somatic mosaicism.

In conclusion, at a detection limit of 0.08% allelic fraction, our amplicon-based *KRAS* mutations sequencing assay applied to a large case-control series of plasma samples showed a limited sensitivity of 21.1% for the detection of pancreatic cancer and was not as specific as anticipated.

We detected 34% of advanced stages and 10% of early stages, suggesting that the limitation in sensitivity is at least partially attributable to the biology of the pancreatic malignancies. Whether reaching a lower threshold of detection for cfDNA mutations could increase the discriminatory performance of the test remains to be assessed. We evaluated whether the combination of the detection of circulating *KRAS* mutations and the plasma CA19-9 levels could improve the detection of pancreatic cancer. We confirm a good sensitivity (90%) but a poor specificity for the CA19-9 plasma levels (64.8%). Combining cfDNA *KRAS* mutations and CA19-9 levels improved the sensitivity to 95% but the overall performance of the combined biomarkers did not significantly improve as compared to CA19-9 alone. However, combining cfDNA *KRAS* mutations could potentially contribute to expanded panels of non-invasive biomarkers involving different tumorigenesis processes and/or different mechanisms of release in the bloodstream, such as protein-based [46], exosome-based [47], methylation-based [48] or RNA-based markers [49], for the risk assessment of the disease.

## MATERIALS AND METHODS

### Study population, sample selection and ethics statement

Samples were selected from a multi-center case-control study conducted in Czech Republic and Slovakia and described in detail elsewhere [50, 51] (Supplementary data).

We conducted this study in two phases, a pilot series where we screened for *KRAS* mutations and measured CA19-9 plasma levels in plasma samples of 96 subjects and a validation series where we extended our initial *KRAS* mutation screening to plasma samples of 967 subjects. For the pilot series, we selected subjects with available plasma and pancreatic tissue (tumor or juice) samples, hence limiting our series to subjects recruited in Czech Republic. We selected all such cases with a histologically-confirmed PDAC diagnosis (N=40) and the 9 subjects diagnosed with chronic pancreatitis (N=9). In addition, we randomly selected 20 healthy controls among 916 with available plasma samples, frequency matched for the 40 PDAC cases on sex, age, tobacco and alcohol consumption. Finally, we selected 27 subjects recruited into the study as pancreatic cancer in first instance, but who subsequently were re-classified as benign neoplasms of the pancreas. For the validation study, we selected all remaining cases with histologically/cytologically confirmed pancreatic cancer (N=421); chronic pancreatitis subjects (N=145); as well as 401 healthy controls among 896, frequency matched for the cancer and chronic pancreatitis subjects on center, sex and age. For pancreatic cancer cases, stage grouping was defined as local, regional, and systemic cancers, based on TNM staging (AJCC 6th edition) when available, and estimation by the clinician when formal TNM staging was not available or not complete.

The study protocol was approved by the institutional review boards of the International Agency for Research on Cancer and all collaborating centers/institutions, and written informed consent was obtained for all participating subjects.

### Isolation of plasma cell-free DNA (cfDNA) and quantification

Peripheral blood from patients was collected in EDTA Vacutainer tubes (Becton Dickinson). Blood samples were processed within 12 h of collection by centrifugation at 2,000g for 10 min and stored frozen in 2mL cryotubes. Circulating DNA (cfDNA) was isolated from 0.6-2.0mL (pilot series; average: 1.4mL) and from 0.3-1.0mL (validation series; average: 0.9mL) plasma with the QIAamp Circulating Nucleic Acid Kit (Qiagen), following manufacturer's instructions [52]. The concentration of purified cfDNA was determined using the Quant-iT™ PicoGreenR dsDNA Assay (Molecular Probes, Invitrogen) PicoGreen® a dilution series of a standard lambda DNA and a Fluoroskan Ascent FL instrument (Thermo Fisher Scientific).

### *KRAS* amplification, library construction and deep sequencing with Ion Torrent PGM

As the size of the cfDNA fragments in cancer patients was recently reported to follow a narrowed-range, unimodal distribution reaching a peak at 166bp [32], primers were designed to amplify exons 2 and 3 so that the amplicon size is < 130bp (79bp and 129bp respectively), covering from codons 4 to 16 (hg19: ch12: 25,398,271 - ch12: 25,398,309) and from codons 51 to 69 (hg19: ch12: 25,380,228 - ch12: 25,380,307), totalling 119 bp excluding primer regions. Forward and reverse primer sequences were 5′-GCCTGCTGAAAATGACTGAA-3′ and 5′-AGCTGTATCGTCAAGGCACT-3′ for the amplification of partial *KRAS* exon 2 and 5′-GCAAGT AGTAATTGATGGAGAAACC-3′ and 5′-TTTATGGCA AATACACAAAGAAAG-3′ for the partial amplification of *KRAS* exon 3. Independent PCR amplifications of the 2 exons were performed using 2ng of cfDNA, 5X AccuStart Buffer, 200 nM forward and reverse primers and 0.04 U/mL of AccuStart HiFi Taq Polymerase (Quanta BioSciences) with the following conditions: 2 min at 94°C, 50 cycles of 30s at 94°C, 30s at 58°C and 40s at 72°C and a final elongation of 5 min at 72°C. Approximately 20% of the PCR products were quantified by Qubit^TM^ dsDNA HS Assay Kit and (Invitrogen) and Qubit^®^ 2.0 fluorometer and 20 ng of exon 2 and 3 were pooled together, purified with Serapure magnetic beads at a final concentration of 2.5X and 28% of isopropanol. Library preparation was done using the NEBNext NEB Next® Fast DNA Library Prep Set for Ion Torrent™ kit (New England Biolabs) with some modifications, where each volume of reagents was reduced by a factor 4. Briefly, 12.5μl of the 20μl purified products were end-repaired in 15μl, and added to 8.6 μl of ligation reaction mix, 0.7μl of the Ion P1 Adapter and 0.7 μl of each Ion Barcode for the ligation step. The barcoded products were purified using Serapure magnetic beads at final concentration of 1.8X, amplified in 25μl and quantified using Qubit quantification system. 40 ng of amplified barcoded products were pooled into a single tube and the cleanup and size selection of pooled libraries (230~250 bp) was performed in a 2% agarose gel and MinElute Gel Extraction Kit (Qiagen). The pool of purified barcoded libraries was quantified using the Qubit quantification system and the assessment of the library quality (molarity and size analysis) was done using the Agilent^®^ High Sensitivity DNA Kit and the Agilent Technologies 2100 Bioanalyzer^TM^ (Agilent Technologies). The pool of purified barcoded libraries was diluted to 280 millions of molecules in 25μl and sequenced with the IonTorrent™ PGM sequencer (Thermo Fisher Scientific) at deep coverage using the Ion OneTouch 200 Template Kit v2 DL and Ion PGM Sequencing 200 Kit v2 with the 316 or 318 chips (Thermo Fisher Scientific), following manufacturer's instructions. Library preparation and sequencing conditions were adapted from previous protocols [43].

### Detection Threshold

Genomic DNA from the cell-line SW480 harboring a hemizygous *KRAS* p.G12V (c.35G>T) mutation was serially diluted into genomic DNA of a human wild-type lymphoblastoid cell-line in order to assess the accuracy and the detection threshold of the Ion Torrent Sequencing for the measurement of the mutant allelic fraction. Mutant abundances were as follows: 100%, 50%, 20%, 10%, 5%, 2%, 1%, 0.5%, 0.2%, 0.1%, 0.05%, 0.02%, 0.01%. Four independent PCR amplifications were done for each serial diluted point and for six wild-type DNA samples to determine the read error rate for that specific genomic position. PCR amplifications from 2ng, library construction and deep sequencing were done following the same protocol as for the cfDNA.

### Measurement of the CA19-9 plasma level

Measurements of CA19-9 were performed on plasma EDTA samples from the pilot study. Analyses were done using an immunoradiometric assay by Beckmann Coulter (Marseille, France). Samples have been randomized through the batches of analyses. We used the clinically accepted cut-off of 37 kU/l for CA19-9 positivity [53].

### Bioinformatics and statistical analyses

We used Needlestack, a variant caller algorithm suitable for the detection of low-abundance mutations [43] (https://github.com/IARCbioinfo/needlestack). The approach is based on the inclusion of sequencing data of a sufficient number of samples to robustly estimate the sequencing error rates at each position considered and for each possible base change. Reads were mapped to the human whole genome and BAM files were generated by the Ion Torrent PGM server using default parameters. Reads with a mapping quality below 20 were excluded from subsequent analysis. At each position and for each candidate variant, sequencing errors are modeled using a robust negative binomial regression [54] to avoid bias of the over-dispersion parameter due to the potential presence of genetic variants. We use a linear link and a zero intercept, and detected variants as being outliers from this error model. We calculated for each sample a p-value for being a variant (outlier from the regression) that we further transformed into q-values to account for multiple testing. *q*-values are reported in Phred scale QVAL=−10 log_10_(*q*-value), and we used a threshold of QVAL>30 to call variants. For each variant, we also calculated the relative variant strand bias defined by:

$$\text{RVSB}=\frac{\max(AO_{p}DP_{m},AO_{m}DP_{p})}{AO_{p}DP_{m}+AO_{m}DP_{p}}$$

where *DP* and *AO* denote respectively the total number of reads and the number of reads matching the candidate variant, with the subscripts *p* and *m* referring to the forward and reverse strands respectively.

## SUPPLEMENTARY FIGURES AND TABLES










