# Supplementary material for: KRAS mutations in blood circulating cell-free DNA: a pancreatic cancer case-control
Source: Oncotarget. 2016 Oct 1;7(48):78827–40. doi: 10.18632/oncotarget.12386 (PMC5346680; doi:10.18632/oncotarget.12386)
Supplement: Supplementary file 1 [file oncotarget-07-78827-s001.pdf]

KRAS mutations in blood circulating cell-free DNA: a pancreatic cancer case-control study

SUPPLEMENTARY FIGURES AND TABLES

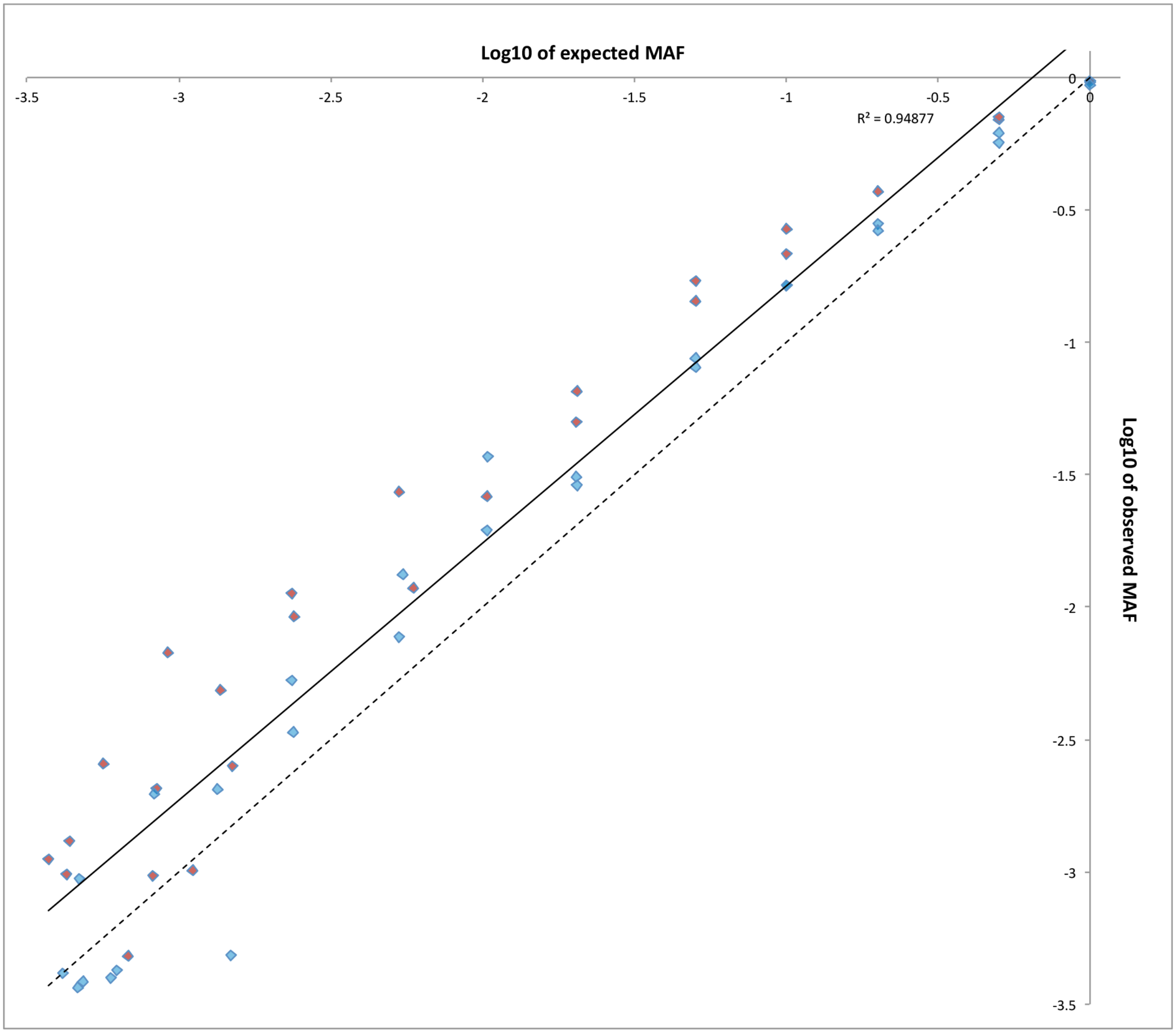

Supplementary Figure S1: Correlation between the observed and expected Mutant Allelic Fractions of serial dilutions of *KRAS* c.35G>T; p.G12V mutated DNA from SW480 cell-line. MAF: Mutant Allelic Fraction defined as LOG10 (AF+1/DP), AF is the Allelic Fraction of the mutant and DP the total Read Depth obtained for the *KRAS* c.35 genomic position using a Ion Torrent PGM 316 Chip (red dots) and a Ion Torrent PGM 318 chip (Blue dots).

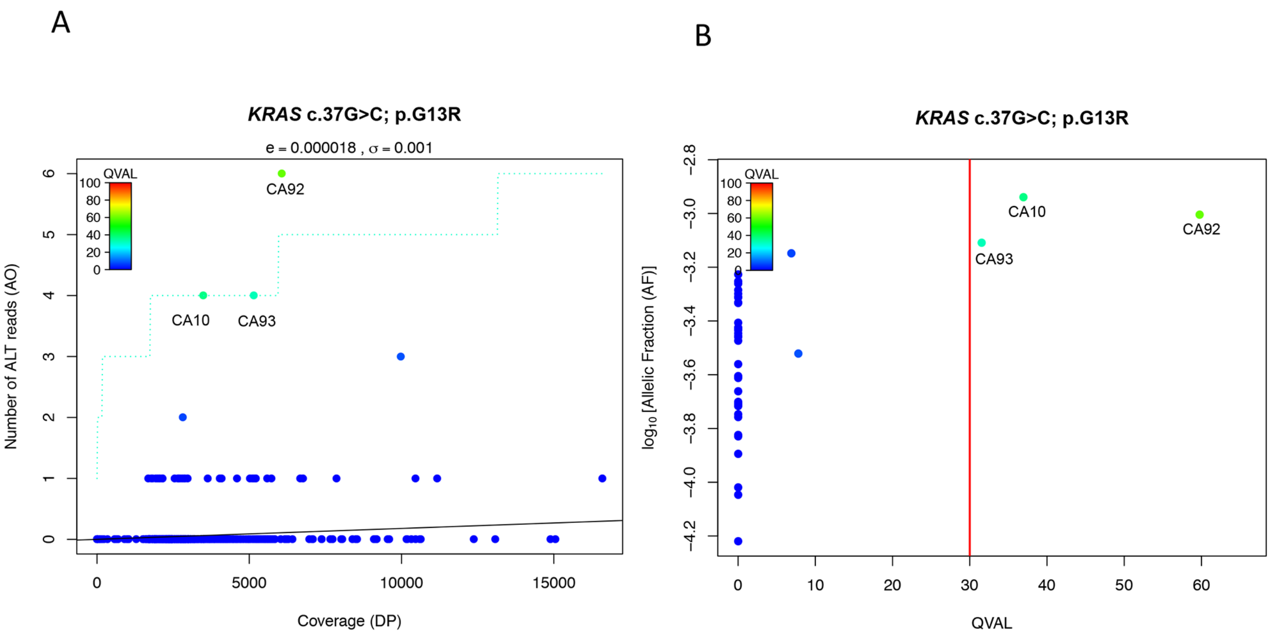

Supplementary Figure S2: Mutation detection of the lowest allelic fraction (*KRAS* c.37G>C; p.G13R) in cfDNA samples of the validation series (N=903) using Needlestack. A. Negative-binomial regression plot at *KRAS* c.37G>C; p.G13R displaying the total number of reads (coverage, DP) and the number of reads matching the candidate variant (AO). Black solid line: Estimated error rate (e) at the c.37 position for G>C base change. Blue dotted line: Detection limit at q-values $<10^{-3}$; >30 in Phred scale (QVAL). Dots above the blue dotted line: Outliers of the regression (QVAL≥30), declared as mutant *KRAS* samples (c.37G>C; p.G13R), e.g. samples CA10, CA92, CA93. Dots below the blue dotted line: Inliers (QVAL<30) declared unmutated at this position for specified base change. **B.** Allelic fraction plot showing the detection limit of 0.08% mutant for sample CA93 ($\log_{10}$ (0.0008) = -3.09).

Supplementary Table S1: Ion Torrent Sequencing and Needlestack analysis of serial dilutions of *KRAS* c.35G>T; p.G12V mutated DNA from SW480 cell-line.

See Supplementary File 1

Supplementary Table S2: List of samples with cfDNA *KRAS* mutations, CA19-9 plasma levels in the pilot series.

See Supplementary File 2

Supplementary Table S3: List of samples with cfDNA *KRAS* mutations in the validation series.

See Supplementary File 3

Supplementary Table S4: List of samples with cfDNA *KRAS* mutations outside of hotspot codons but reported in COSMIC.

See Supplementary File 4
