# Supplementary material for: KRAS mutations in blood circulating cell-free DNA: a pancreatic cancer case-control
Source: Oncotarget. 2016 Oct 1;7(48):78827–40. doi: 10.18632/oncotarget.12386 (PMC5346680; doi:10.18632/oncotarget.12386)
Supplement: Supplementary file 2 [file oncotarget-07-78827-s002.docx]

Supplementary Table S1. Ion Torrent Sequencing and Needlestack analysis of serial dilutions of *KRAS* c.35G>T; p.G12V mutated DNA from SW480 cell-line

| \| \| Serial dilution SW480 p.G12V DNA \| Expected AF^a^ (%) \| Observed AF^a^ (%) \| Read Depth \| Mutated reads \| RVSB^b^ \| QVAL (Phred scale q-value) \| \| --- \| --- \| --- \| --- \| --- \| --- \| --- \| \| SW480_100_316chip \| 100 \| 96.25 \| 4189 \| 4032 \| 0.51 \| 2809.99 \| \| SW480_100_316chip_rep \| 100 \| 96.97 \| 3098 \| 3004 \| 0.50 \| 2760.52 \| \| SW480_100_318chip \| 100 \| 96.60 \| 4205 \| 4062 \| 0.50 \| Infinite \| \| SW480_100_318chip_rep \| 100 \| 93.55 \| 1612 \| 1508 \| 0.51 \| Infinite \| \| SW480_50_316chip \| 50 \| 69.31 \| 3838 \| 2660 \| 0.51 \| 2013.93 \| \| SW480_50_316chip_rep \| 50 \| 70.97 \| 2918 \| 2071 \| 0.52 \| 2011.92 \| \| SW480_50_318chip \| 50 \| 61.83 \| 2845 \| 1759 \| 0.52 \| Infinite \| \| SW480_50_318chip_rep \| 50 \| 56.58 \| 2462 \| 1393 \| 0.52 \| Infinite \| \| SW480_20_316chip \| 20 \| 36.97 \| 3649 \| 1349 \| 0.55 \| 1073.05 \| \| SW480_20_316chip_rep \| 20 \| 37.15 \| 3295 \| 1224 \| 0.53 \| 1069.19 \| \| SW480_20_318chip \| 20 \| 27.95 \| 2716 \| 759 \| 0.51 \| Infinite \| \| SW480_20_318chip_rep \| 20 \| 26.42 \| 2544 \| 672 \| 0.54 \| Infinite \| \| SW480_10_316chip \| 10 \| 21.51 \| 2906 \| 625 \| 0.59 \| 614.17 \| \| SW480_10_316chip_rep \| 10 \| 26.70 \| 2371 \| 633 \| 0.57 \| 743.47 \| \| SW480_10_318chip \| 10 \| 16.33 \| 2793 \| 456 \| 0.54 \| 2574.72 \| \| SW480_10_318chip_rep \| 10 \| 16.37 \| 2273 \| 372 \| 0.54 \| 2346.43 \| \| SW480_5_316chip \| 5 \| 17.01 \| 2869 \| 488 \| 0.52 \| 486.37 \| \| SW480_5_316chip_rep \| 5 \| 14.25 \| 2885 \| 411 \| 0.57 \| 408.64 \| \| SW480_5_318chip \| 5 \| 8.03 \| 2577 \| 207 \| 0.59 \| 1211.93 \| \| SW480_5_318chip_rep \| 5 \| 8.65 \| 3641 \| 315 \| 0.56 \| 1518.07 \| \| SW480_2_316chip \| 2 \| 4.97 \| 2476 \| 123 \| 0.55 \| 142.14 \| \| SW480_2_316chip_rep \| 2 \| 6.49 \| 2328 \| 151 \| 0.60 \| 183.27 \| \| SW480_2_318chip \| 2 \| 2.84 \| 2398 \| 68 \| 0.52 \| 402.64 \| \| SW480_2_318chip_rep \| 2 \| 3.07 \| 2903 \| 89 \| 0.51 \| 477.56 \| \| SW480_1_316chip \| 1 \| 2.59 \| 2861 \| 74 \| 0.56 \| 76.45 \| \| SW480_1_316chip_rep \| 1 \| Failed \| Failed \| Failed \| Failed \| Failed \| \| SW480_1_318chip \| 1 \| 3.66 \| 2378 \| 87 \| 0.57 \| 521.93 \| \| SW480_1_318chip_rep \| 1 \| 1.91 \| 2876 \| 55 \| 0.55 \| 290.74 \| \| SW480_0.5_316chip \| 0.5 \| 2.69 \| 3238 \| 87 \| 0.59 \| 80.10 \| \| SW480_0.5_3016chip_rep \| 0.5 \| 1.09 \| 1104 \| 12 \| 0.55 \| 27.26 \| \| SW480_0.5_318chip \| 0.5 \| 1.28 \| 2117 \| 27 \| 0.58 \| 161.80 \| \| SW480_0.5_318chip_rep \| 0.5 \| 0.74 \| 3249 \| 24 \| 0.58 \| 109.47 \| \| SW480_0.2_316chip \| 0.2 \| 1.09 \| 2846 \| 31 \| 0.69 \| 32.10 \| \| SW480_0.2_316chip_rep \| 0.2 \| 0.88 \| 2498 \| 22 \| 0.65 \| 25.72 \| \| SW480_0.2_318chip \| 0.2 \| 0.30 \| 2670 \| 8 \| 0.50 \| 32.54 \| \| SW480_0.2_318chip_rep \| 0.2 \| 0.49 \| 2831 \| 14 \| 0.67 \| 63.97 \| \| SW480_0.1_316chip \| 0.1 \| 0.45 \| 2672 \| 12 \| 0.62 \| 12.82 \| \| SW480_0.1_316chip_rep \| 0.1 \| 0.20 \| 1994 \| 4 \| 0.88 \| 4.34 \| \| SW480_0.1_318chip \| 0.1 \| 0.00 \| 2066 \| 0 \| NA \| 0.00 \| \| SW480_0.1_318chip_rep \| 0.1 \| 0.17 \| 2925 \| 5 \| 0.56 \| 15.76 \| \| SW480_0.05_316chip \| 0.05 \| 0.17 \| 2904 \| 5 \| 0.50 \| 4.06 \| \| SW480_0.05_3016chip_rep \| 0.05 \| 0.63 \| 2376 \| 15 \| 0.52 \| 18.25 \| \| SW480_0.05_318chip \| 0.05 \| 0.16 \| 3036 \| 5 \| 1.00 \| 15.24 \| \| SW480_0.05_318chip_rep \| 0.05 \| 0.06 \| 3106 \| 2 \| 0.70 \| 1.50 \| \| SW480_0.02_316chip \| 0.02 \| 0.22 \| 2736 \| 6 \| 0.63 \| 5.33 \| \| SW480_0.02_316chip_rep \| 0.02 \| 0.00 \| 2074 \| 0 \| NA \| 0.00 \| \| SW480_0.02_318chip \| 0.02 \| 0.00 \| 2517 \| 0 \| NA \| 0.00 \| \| SW480_0.02_318chip_rep \| 0.02 \| 0.00 \| 2350 \| 0 \| NA \| 0.00 \| \| SW480_0.01_316chip \| 0.01 \| 0.07 \| 3071 \| 2 \| 0.61 \| 1.19 \| \| SW480_0.01_316chip_rep \| 0.01 \| 0.00 \| 991 \| 0 \| NA \| 0.00 \| \| SW480_0.01_318chip \| 0.01 \| 0.00 \| 2602 \| 0 \| NA \| 0.00 \| \| SW480_0.01_318chip_rep \| 0.01 \| 0.00 \| 2737 \| 0 \| NA \| 0.00 \| \| SW480_0_316chip \| 0 \| 0.07 \| 2688 \| 2 \| 0.61 \| 1.19 \| \| SW480_0_316chip_rep \| 0 \| 0.09 \| 2293 \| 2 \| 0.60 \| 1.25 \| \| SW480_0_318chip \| 0 \| 0.00 \| 2410 \| 0 \| NA \| 0.00 \| \| SW480_0_318chip_rep \| 0 \| 0.05 \| 2126 \| 1 \| 1.00 \| 0.00 \| \| \| --- \| --- \| --- \| --- \| --- \| --- \| --- \| --- \| --- \| --- \| --- \| --- \| --- \| --- \| --- \| --- \| --- \| --- \| --- \| --- \| --- \| --- \| --- \| --- \| --- \| --- \| --- \| --- \| --- \| --- \| --- \| --- \| --- \| --- \| --- \| --- \| --- \| --- \| --- \| --- \| --- \| --- \| --- \| --- \| --- \| --- \| --- \| --- \| --- \| --- \| --- \| --- \| --- \| --- \| --- \| --- \| --- \| --- \| --- \| --- \| --- \| --- \| --- \| --- \| --- \| --- \| --- \| --- \| --- \| --- \| --- \| --- \| --- \| --- \| --- \| --- \| --- \| --- \| --- \| --- \| --- \| --- \| --- \| --- \| --- \| --- \| --- \| --- \| --- \| --- \| --- \| --- \| --- \| --- \| --- \| --- \| --- \| --- \| --- \| --- \| --- \| --- \| --- \| --- \| --- \| --- \| --- \| --- \| --- \| --- \| --- \| --- \| --- \| --- \| --- \| --- \| --- \| --- \| --- \| --- \| --- \| --- \| --- \| --- \| --- \| --- \| --- \| --- \| --- \| --- \| --- \| --- \| --- \| --- \| --- \| --- \| --- \| --- \| --- \| --- \| --- \| --- \| --- \| --- \| --- \| --- \| --- \| --- \| --- \| --- \| --- \| --- \| --- \| --- \| --- \| --- \| --- \| --- \| --- \| --- \| --- \| --- \| --- \| --- \| --- \| --- \| --- \| --- \| --- \| --- \| --- \| --- \| --- \| --- \| --- \| --- \| --- \| --- \| --- \| --- \| --- \| --- \| --- \| --- \| --- \| --- \| --- \| --- \| --- \| --- \| --- \| --- \| --- \| --- \| --- \| --- \| --- \| --- \| --- \| --- \| --- \| --- \| --- \| --- \| --- \| --- \| --- \| --- \| --- \| --- \| --- \| --- \| --- \| --- \| --- \| --- \| --- \| --- \| --- \| --- \| --- \| --- \| --- \| --- \| --- \| --- \| --- \| --- \| --- \| --- \| --- \| --- \| --- \| --- \| --- \| --- \| --- \| --- \| --- \| --- \| --- \| --- \| --- \| --- \| --- \| --- \| --- \| --- \| --- \| --- \| --- \| --- \| --- \| --- \| --- \| --- \| --- \| --- \| --- \| --- \| --- \| --- \| --- \| --- \| --- \| --- \| --- \| --- \| --- \| --- \| --- \| --- \| --- \| --- \| --- \| --- \| --- \| --- \| --- \| --- \| --- \| --- \| --- \| --- \| --- \| --- \| --- \| --- \| --- \| --- \| --- \| --- \| --- \| --- \| --- \| --- \| --- \| --- \| --- \| --- \| --- \| --- \| --- \| --- \| --- \| --- \| --- \| --- \| --- \| --- \| --- \| --- \| --- \| --- \| --- \| --- \| --- \| --- \| --- \| --- \| --- \| --- \| --- \| --- \| --- \| --- \| --- \| --- \| --- \| --- \| --- \| --- \| --- \| --- \| --- \| --- \| --- \| --- \| --- \| --- \| --- \| --- \| --- \| --- \| --- \| --- \| --- \| --- \| --- \| --- \| --- \| --- \| --- \| --- \| --- \| --- \| --- \| --- \| --- \| --- \| --- \| --- \| --- \| --- \| --- \| --- \| --- \| --- \| --- \| --- \| --- \| --- \| --- \| --- \| --- \| --- \| --- \| --- \| --- \| --- \| --- \| --- \| --- \| --- \| --- \| --- \| --- \| --- \| --- \| --- \| --- \| --- \| --- \| --- \| --- \| --- \| --- \| --- \| --- \| --- \| |
| --- | --- | --- | --- | --- | --- | --- | --- | --- | --- | --- | --- | --- | --- | --- | --- | --- | --- | --- | --- | --- | --- | --- | --- | --- | --- | --- | --- | --- | --- | --- | --- | --- | --- | --- | --- | --- | --- | --- | --- | --- | --- | --- | --- | --- | --- | --- | --- | --- | --- | --- | --- | --- | --- | --- | --- | --- | --- | --- | --- | --- | --- | --- | --- | --- | --- | --- | --- | --- | --- | --- | --- | --- | --- | --- | --- | --- | --- | --- | --- | --- | --- | --- | --- | --- | --- | --- | --- | --- | --- | --- | --- | --- | --- | --- | --- | --- | --- | --- | --- | --- | --- | --- | --- | --- | --- | --- | --- | --- | --- | --- | --- | --- | --- | --- | --- | --- | --- | --- | --- | --- | --- | --- | --- | --- | --- | --- | --- | --- | --- | --- | --- | --- | --- | --- | --- | --- | --- | --- | --- | --- | --- | --- | --- | --- | --- | --- | --- | --- | --- | --- | --- | --- | --- | --- | --- | --- | --- | --- | --- | --- | --- | --- | --- | --- | --- | --- | --- | --- | --- | --- | --- | --- | --- | --- | --- | --- | --- | --- | --- | --- | --- | --- | --- | --- | --- | --- | --- | --- | --- | --- | --- | --- | --- | --- | --- | --- | --- | --- | --- | --- | --- | --- | --- | --- | --- | --- | --- | --- | --- | --- | --- | --- | --- | --- | --- | --- | --- | --- | --- | --- | --- | --- | --- | --- | --- | --- | --- | --- | --- | --- | --- | --- | --- | --- | --- | --- | --- | --- | --- | --- | --- | --- | --- | --- | --- | --- | --- | --- | --- | --- | --- | --- | --- | --- | --- | --- | --- | --- | --- | --- | --- | --- | --- | --- | --- | --- | --- | --- | --- | --- | --- | --- | --- | --- | --- | --- | --- | --- | --- | --- | --- | --- | --- | --- | --- | --- | --- | --- | --- | --- | --- | --- | --- | --- | --- | --- | --- | --- | --- | --- | --- | --- | --- | --- | --- | --- | --- | --- | --- | --- | --- | --- | --- | --- | --- | --- | --- | --- | --- | --- | --- | --- | --- | --- | --- | --- | --- | --- | --- | --- | --- | --- | --- | --- | --- | --- | --- | --- | --- | --- | --- | --- | --- | --- | --- | --- | --- | --- | --- | --- | --- | --- | --- | --- | --- | --- | --- | --- | --- | --- | --- | --- | --- | --- | --- | --- | --- | --- | --- | --- | --- | --- | --- | --- | --- | --- | --- | --- | --- | --- | --- | --- | --- | --- | --- | --- | --- | --- | --- | --- | --- | --- | --- | --- | --- | --- | --- | --- | --- | --- |

^a^AF : Allelic Fraction

^b^RVSB: Relative Variant Strand Biais
