# Supplementary material for: KRAS mutations in blood circulating cell-free DNA: a pancreatic cancer case-control
Source: Oncotarget. 2016 Oct 1;7(48):78827–40. doi: 10.18632/oncotarget.12386 (PMC5346680; doi:10.18632/oncotarget.12386)
Supplement: Supplementary file 3 [file oncotarget-07-78827-s003.docx]

Supplementary Table S2. List of samples with cfDNA *KRAS* mutations, CA19-9 plasma levels in the pilot series

| \| Status \| Sample ID \| Stage \| QVAL^a^ \| Read Depth \| Mutated Reads \| RVSB^b^ \| HGVS cDNA \| HGVS protein \| Mutation type \| AF^c^ (%) \| Reported in COSMIC (PDAC) \| Reported in COSMIC (all cancer sites) \| CA-19-9 (kU/l) \| \| --- \| --- \| --- \| --- \| --- \| --- \| --- \| --- \| --- \| --- \| --- \| --- \| --- \| --- \| \| Benign pancreatic neoplasm \| BE1 \| NA \| 979.09 \| 2216 \| 41 \| 0.67 \| c.34G>C \| p.G12R \| Missense \| 1.85 \| YES \| YES \| 797 \| \| Benign pancreatic neoplasm \| BE2 \| NA \| 42.66 \| 3865 \| 5 \| 0.51 \| c.31G>C \| p.A11P \| Missense \| 0.13 \| NO \| YES \| 10 \| \| PDAC case \| CA1 \| Systemic \| 41.23 \| 3301 \| 19 \| 0.67 \| c.28G>A \| p.G10R \| Missense \| 0.58 \| NO \| YES \| 365 \| \| PDAC case \| CA1 \| Systemic \| 34.05 \| 2746 \| 27 \| 0.54 \| c.14A>G \| p.K5R \| Missense \| 0.98 \| NO \| YES \| 365 \| \| PDAC case \| CA2 \| Systemic \| Infinite \| 2778 \| 987 \| 0.55 \| c.35G>A \| p.G12D \| Missense \| 35.53 \| YES \| YES \| 1111 \| \| PDAC case \| CA3 \| Systemic \| 77.48 \| 5654 \| 48 \| 0.62 \| c.35G>A \| p.G12D \| Missense \| 0.85 \| YES \| YES \| 22 \| \| PDAC case \| CA4 \| Systemic \| 89.71 \| 6806 \| 195 \| 0.61 \| c.35G>T \| p.G12V \| Missense \| 2.87 \| YES \| YES \| 2344 \| \| PDAC case \| CA5 \| Systemic \| 1290.67 \| 3244 \| 55 \| 0.56 \| c.34G>C \| p.G12R \| Missense \| 1.70 \| YES \| YES \| 25 \| \| PDAC case \| CA6 \| Regional \| 71.32 \| 4743 \| 40 \| 0.64 \| c.35G>A \| p.G12D \| Missense \| 0.84 \| YES \| YES \| 605 \| \| PDAC case \| CA7 \| Systemic \| 44.01 \| 3048 \| 32 \| 0.51 \| c.14A>G \| p.K5R \| Missense \| 1.05 \| NO \| YES \| 283 \| \| PDAC case \| CA8 \| Local \| 231.51 \| 4637 \| 87 \| 0.51 \| c.35G>A \| p.G12D \| Missense \| 1.88 \| YES \| YES \| 242 \| \| PDAC case \| CA9 \| Systemic \| 168.81 \| 3673 \| 59 \| 0.55 \| c.35G>A \| p.G12D \| Missense \| 1.61 \| YES \| YES \| 65 \| |
| --- | --- | --- | --- | --- | --- | --- | --- | --- | --- | --- | --- | --- | --- | --- | --- | --- | --- | --- | --- | --- | --- | --- | --- | --- | --- | --- | --- | --- | --- | --- | --- | --- | --- | --- | --- | --- | --- | --- | --- | --- | --- | --- | --- | --- | --- | --- | --- | --- | --- | --- | --- | --- | --- | --- | --- | --- | --- | --- | --- | --- | --- | --- | --- | --- | --- | --- | --- | --- | --- | --- | --- | --- | --- | --- | --- | --- | --- | --- | --- | --- | --- | --- | --- | --- | --- | --- | --- | --- | --- | --- | --- | --- | --- | --- | --- | --- | --- | --- | --- | --- | --- | --- | --- | --- | --- | --- | --- | --- | --- | --- | --- | --- | --- | --- | --- | --- | --- | --- | --- | --- | --- | --- | --- | --- | --- | --- | --- | --- | --- | --- | --- | --- | --- | --- | --- | --- | --- | --- | --- | --- | --- | --- | --- | --- | --- | --- | --- | --- | --- | --- | --- | --- | --- | --- | --- | --- | --- | --- | --- | --- | --- | --- | --- | --- | --- | --- | --- | --- | --- | --- | --- | --- | --- | --- | --- | --- | --- | --- | --- | --- | --- | --- |

^a^QVAL: Phred scale q-value; ^b^RVSB: Relative Variant Strand Biais; ^c^AF : Allelic Fraction
