# Supplementary material for: KRAS mutations in blood circulating cell-free DNA: a pancreatic cancer case-control
Source: Oncotarget. 2016 Oct 1;7(48):78827–40. doi: 10.18632/oncotarget.12386 (PMC5346680; doi:10.18632/oncotarget.12386)
Supplement: Supplementary file 4 [file oncotarget-07-78827-s004.docx]

Supplementary Table S3. List of samples with cfDNA *KRAS* mutations in the validation series

| \| \| **Pancreatic cancer cases with a single KRAS cfDNA mutation at hotspot codon 12, 13 or 61 reported in pancreas** \| \| \| \| \| \| \| \| \| \|  \|  \|  \|  \|  \| \| --- \| --- \| --- \| --- \| --- \| --- \| --- \| --- \| --- \| --- \| --- \| --- \| --- \| --- \| --- \| \|  \| Status \| Sample ID \| Stage \| QVAL^a^ \| Read Depth \| Mutated Reads \| RVSB^b^ \|  \| HGVS cDNA \| HGVS protein \| Mutation type \| AF^c^ (%) \|  \| comments \| \|  \| Unknown/Missing \| CA10 \| Regional \| 36.93 \| 3492 \| 4 \| 0.73 \|  \| c.37G>C \| p.G13R \| missense \| 0.11 \|  \| NA \| \|  \| Unknown/Missing \| CA11 \| Unknown \| 33.76 \| 2050 \| 4 \| 1.00 \|  \| c.34G>C \| p.G12R \| missense \| 0.20 \|  \| NA \| \|  \| PDAC \| CA12 \| Systemic \| 42.63 \| 7057 \| 17 \| 0.75 \|  \| c.183A>C \| p.Q61H \| missense \| 0.24 \|  \| NA \| \|  \| PDAC \| CA13 \| Systemic \| 74.63 \| 3209 \| 8 \| 0.81 \|  \| c.34G>C \| p.G12R \| missense \| 0.25 \|  \| NA \| \|  \| PDAC \| CA14 \| Unknown \| 53.33 \| 5255 \| 14 \| 0.65 \|  \| c.34G>T \| p.G12C \| missense \| 0.27 \|  \| NA \| \|  \| Unknown/Missing \| CA15 \| Unknown \| 50.33 \| 1761 \| 5 \| 0.72 \|  \| c.34G>C \| p.G12R \| missense \| 0.28 \|  \| NA \| \|  \| PDAC \| CA16 \| Systemic \| 39.91 \| 3027 \| 10 \| 0.84 \|  \| c.35G>T \| p.G12V \| missense \| 0.33 \|  \| NA \| \|  \| PDAC \| CA17 \| Systemic \| 37.79 \| 2179 \| 8 \| 0.50 \|  \| c.35G>T \| p.G12V \| missense \| 0.37 \|  \| NA \| \|  \| PDAC \| CA18 \| Regional \| 65.80 \| 4900 \| 20 \| 0.72 \|  \| c.35G>T \| p.G12V \| missense \| 0.41 \|  \| NA \| \|  \| Unknown/Missing \| CA19 \| Unknown \| 46.56 \| 2449 \| 10 \| 0.83 \|  \| c.35G>T \| p.G12V \| missense \| 0.41 \|  \| NA \| \|  \| PDAC \| CA20 \| Regional \| 197.32 \| 4722 \| 20 \| 0.51 \|  \| c.34G>C \| p.G12R \| missense \| 0.42 \|  \| NA \| \|  \| Unknown/Missing \| CA21 \| Unknown \| 55.18 \| 3069 \| 13 \| 0.56 \|  \| c.35G>T \| p.G12V \| missense \| 0.42 \|  \| NA \| \|  \| PDAC \| CA22 \| Systemic \| 124.39 \| 2441 \| 11 \| 1.00 \|  \| c.34G>C \| p.G12R \| missense \| 0.45 \|  \| NA \| \|  \| PDAC \| CA23 \| Regional \| 124.39 \| 2438 \| 11 \| 0.52 \|  \| c.34G>C \| p.G12R \| missense \| 0.45 \|  \| NA \| \|  \| PDAC \| CA24 \| Systemic \| 80.96 \| 4028 \| 21 \| 0.59 \|  \| c.35G>T \| p.G12V \| missense \| 0.52 \|  \| NA \| \|  \| Unknown/Missing \| CA25 \| Regional \| 37.36 \| 6270 \| 39 \| 0.56 \|  \| c.35G>A \| p.G12D \| missense \| 0.62 \|  \| NA \| \|  \| PDAC \| CA26 \| Systemic \| 295.53 \| 4496 \| 28 \| 0.75 \|  \| c.34G>C \| p.G12R \| missense \| 0.62 \|  \| NA \| \|  \| PDAC \| CA27 \| Unknown \| 35.95 \| 5608 \| 35 \| 0.58 \|  \| c.35G>A \| p.G12D \| missense \| 0.62 \|  \| NA \| \|  \| PDAC \| CA28 \| Systemic \| 98.71 \| 3786 \| 24 \| 0.61 \|  \| c.35G>T \| p.G12V \| missense \| 0.63 \|  \| NA \| \|  \| PDAC \| CA29 \| Systemic \| 318.38 \| 4517 \| 30 \| 0.61 \|  \| c.34G>C \| p.G12R \| missense \| 0.66 \|  \| NA \| \|  \| PDAC \| CA30 \| Systemic \| 263.50 \| 4917 \| 33 \| 0.73 \|  \| c.34G>T \| p.G12C \| missense \| 0.67 \|  \| NA \| \|  \| PDAC \| CA31 \| Regional \| 115.22 \| 4122 \| 29 \| 0.50 \|  \| c.35G>T \| p.G12V \| missense \| 0.70 \|  \| NA \| \|  \| Endocrine \| CA32 \| Regional \| 148.33 \| 6481 \| 49 \| 0.50 \|  \| c.35G>T \| p.G12V \| missense \| 0.76 \|  \| NA \| \|  \| PDAC \| CA33 \| Systemic \| 47.49 \| 3958 \| 31 \| 0.58 \|  \| c.35G>A \| p.G12D \| missense \| 0.78 \|  \| NA \| \|  \| Unknown/Missing \| CA34 \| Systemic \| 75.11 \| 10234 \| 85 \| 0.54 \|  \| c.35G>A \| p.G12D \| missense \| 0.83 \|  \| NA \| \|  \| Unknown/Missing \| CA35 \| Systemic \| 404.25 \| 4438 \| 37 \| 0.53 \|  \| c.34G>C \| p.G12R \| missense \| 0.83 \|  \| NA \| \|  \| Unknown/Missing \| CA36 \| Regional \| 150.94 \| 4128 \| 37 \| 0.66 \|  \| c.35G>T \| p.G12V \| missense \| 0.90 \|  \| NA \| \|  \| Unknown/Missing \| CA37 \| Unknown \| 87.98 \| 5378 \| 56 \| 0.69 \|  \| c.35G>A \| p.G12D \| missense \| 1.04 \|  \| NA \| \|  \| PDAC \| CA38 \| Systemic \| 68.76 \| 2925 \| 31 \| 0.52 \|  \| c.35G>A \| p.G12D \| missense \| 1.06 \|  \| NA \| \|  \| PDAC \| CA39 \| Regional \| 87.01 \| 4177 \| 46 \| 0.60 \|  \| c.35G>A \| p.G12D \| missense \| 1.10 \|  \| NA \| \|  \| PDAC \| CA40 \| Systemic \| 232.91 \| 5198 \| 64 \| 0.60 \|  \| c.35G>T \| p.G12V \| missense \| 1.23 \|  \| NA \| \|  \| PDAC \| CA41 \| Regional \| 241.65 \| 5046 \| 65 \| 0.61 \|  \| c.35G>T \| p.G12V \| missense \| 1.29 \|  \| NA \| \|  \| PDAC \| CA42 \| Regional \| 124.87 \| 4432 \| 61 \| 0.56 \|  \| c.35G>A \| p.G12D \| missense \| 1.38 \|  \| NA \| \|  \| PDAC \| CA43 \| Systemic \| 251.20 \| 3537 \| 54 \| 0.60 \|  \| c.35G>T \| p.G12V \| missense \| 1.53 \|  \| NA \| \|  \| PDAC \| CA44 \| Systemic \| 136.15 \| 3695 \| 57 \| 0.51 \|  \| c.35G>A \| p.G12D \| missense \| 1.54 \|  \| NA \| \|  \| Unknown/Missing \| CA45 \| Unknown \| 296.80 \| 4755 \| 76 \| 0.57 \|  \| c.35G>T \| p.G12V \| missense \| 1.60 \|  \| NA \| \|  \| Unknown/Missing \| CA46 \| Systemic \| 386.56 \| 9266 \| 156 \| 0.59 \|  \| c.35G>T \| p.G12V \| missense \| 1.68 \|  \| NA \| \|  \| PDAC \| CA47 \| Systemic \| 671.66 \| 4529 \| 89 \| 0.69 \|  \| c.180_181TC>GA \| p.Q61K \| missense \| 1.69 \|  \| mutations on same reads \| \|  \| PDAC \| CA48 \| Systemic \| 274.32 \| 977 \| 17 \| 0.62 \|  \| c.34G>C \| p.G12R \| missense \| 1.74 \|  \| NA \| \|  \| PDAC \| CA49 \| Systemic \| 199.13 \| 5283 \| 97 \| 0.55 \|  \| c.35G>A \| p.G12D \| missense \| 1.84 \|  \| NA \| \|  \| Unknown/Missing \| CA50 \| Unknown \| 364.15 \| 2788 \| 67 \| 0.51 \|  \| c.35G>T \| p.G12V \| missense \| 2.40 \|  \| NA \| \|  \| PDAC \| CA51 \| Systemic \| 348.70 \| 9158 \| 230 \| 0.57 \|  \| c.35G>A \| p.G12D \| missense \| 2.51 \|  \| NA \| \|  \| PDAC \| CA52 \| Systemic \| 259.09 \| 3456 \| 88 \| 0.54 \|  \| c.35G>A \| p.G12D \| missense \| 2.55 \|  \| NA \| \|  \| PDAC \| CA53 \| Systemic \| 564.49 \| 4128 \| 129 \| 0.61 \|  \| c.35G>T \| p.G12V \| missense \| 3.13 \|  \| NA \| \|  \| Unknown/Missing \| CA54 \| Unknown \| 415.62 \| 5461 \| 178 \| 0.55 \|  \| c.35G>A \| p.G12D \| missense \| 3.26 \|  \| NA \| \|  \| PDAC \| CA55 \| Systemic \| 566.71 \| 3642 \| 120 \| 0.59 \|  \| c.35G>T \| p.G12V \| missense \| 3.29 \|  \| NA \| \|  \| Unknown/Missing \| CA56 \| Unknown \| 607.23 \| 8255 \| 278 \| 0.53 \|  \| c.182A>T \| p.Q61L \| missense \| 3.37 \|  \| NA \| \|  \| PDAC \| CA57 \| Systemic \| 677.13 \| 5043 \| 175 \| 0.51 \|  \| c.35G>T \| p.G12V \| missense \| 3.47 \|  \| NA \| \|  \| PDAC \| CA58 \| Regional \| 552.67 \| 12392 \| 434 \| 0.55 \|  \| c.35G>A \| p.G12D \| missense \| 3.50 \|  \| NA \| \|  \| PDAC \| CA59 \| Regional \| 2031.40 \| 5034 \| 178 \| 0.64 \|  \| c.34G>C \| p.G12R \| missense \| 3.54 \|  \| NA \| \|  \| Unknown/Missing \| CA60 \| Unknown \| 530.61 \| 2277 \| 82 \| 0.57 \|  \| c.183A>T \| p.Q61H \| missense \| 3.60 \|  \| NA \| \|  \| PDAC \| CA61 \| Systemic \| 445.04 \| 3117 \| 128 \| 0.51 \|  \| c.35G>A \| p.G12D \| missense \| 4.11 \|  \| NA \| \|  \| PDAC \| CA62 \| Systemic \| 669.93 \| 5603 \| 273 \| 0.57 \|  \| c.35G>A \| p.G12D \| missense \| 4.87 \|  \| NA \| \|  \| PDAC \| CA63 \| Regional \| 729.57 \| 5775 \| 301 \| 0.61 \|  \| c.35G>A \| p.G12D \| missense \| 5.21 \|  \| NA \| \|  \| PDAC \| CA64 \| Systemic \| 1078.48 \| 7468 \| 519 \| 0.53 \|  \| c.35G>A \| p.G12D \| missense \| 6.95 \|  \| NA \| \|  \| Other ductal \| CA65 \| Regional \| 1081.29 \| 2089 \| 146 \| 0.56 \|  \| c.183A>T \| p.Q61H \| missense \| 6.99 \|  \| NA \| \|  \| Unknown/Missing \| CA66 \| Unknown \| 1137.96 \| 2395 \| 187 \| 0.62 \|  \| c.35G>T \| p.G12V \| missense \| 7.81 \|  \| NA \| \|  \| Unknown/Missing \| CA67 \| Unknown \| Infinite \| 5578 \| 464 \| 0.61 \|  \| c.34G>T \| p.G12C \| missense \| 8.32 \|  \| NA \| \|  \| Other ductal \| CA68 \| Regional \| 1787.23 \| 5279 \| 468 \| 0.56 \|  \| c.35G>T \| p.G12V \| missense \| 8.87 \|  \| NA \| \|  \| PDAC \| CA69 \| Systemic \| 2103.16 \| 7412 \| 695 \| 0.55 \|  \| c.35G>T \| p.G12V \| missense \| 9.38 \|  \| NA \| \|  \| PDAC \| CA70 \| Systemic \| 1711.59 \| 5260 \| 611 \| 0.57 \|  \| c.35G>A \| p.G12D \| missense \| 11.62 \|  \| NA \| \|  \| Unknown/Missing \| CA71 \| Unknown \| Infinite \| 6052 \| 705 \| 0.52 \|  \| c.34G>C \| p.G12R \| missense \| 11.65 \|  \| NA \| \|  \| PDAC \| CA72 \| Regional \| 1649.05 \| 3758 \| 467 \| 0.56 \|  \| c.35G>A \| p.G12D \| missense \| 12.43 \|  \| NA \| \|  \| Unknown/Missing \| CA73 \| Unknown \| Infinite \| 10065 \| 1836 \| 0.57 \|  \| c.35G>T \| p.G12V \| missense \| 18.24 \|  \| NA \| \|  \| Unknown/Missing \| CA74 \| Regional \| 3072.62 \| 5603 \| 1113 \| 0.58 \|  \| c.35G>A \| p.G12D \| missense \| 19.86 \|  \| NA \| \|  \| Other ductal \| CA75 \| Systemic \| Infinite \| 4262 \| 1120 \| 0.52 \|  \| c.34G>C \| p.G12R \| missense \| 26.28 \|  \| NA \| \|  \| PDAC \| CA76 \| Systemic \| Infinite \| 6295 \| 1973 \| 0.54 \|  \| c.35G>A \| p.G12D \| missense \| 31.34 \|  \| NA \| \|  \| PDAC \| CA77 \| Systemic \| Infinite \| 5816 \| 1998 \| 0.53 \|  \| c.35G>T \| p.G12V \| missense \| 34.35 \|  \| NA \| \|  \| Unknown/Missing \| CA78 \| Local \| Infinite \| 6627 \| 3147 \| 0.51 \|  \| c.182A>G \| p.Q61R \| missense \| 47.49 \|  \| NA \| \|  \| PDAC \| CA79 \| Systemic \| Infinite \| 4286 \| 2586 \| 0.50 \|  \| c.34G>C \| p.G12R \| missense \| 60.34 \|  \| NA \| \|  \| Other ductal \| CA80 \| Regional \| Infinite \| 8493 \| 6254 \| 0.52 \|  \| c.35G>A \| p.G12D \| missense \| 73.64 \|  \| NA \| \|  \| PDAC \| CA81 \| Systemic \| Infinite \| 7612 \| 5941 \| 0.50 \|  \| c.34G>C \| p.G12R \| missense \| 78.05 \|  \| NA \| \|  \|  \|  \|  \|  \|  \|  \|  \|  \|  \|  \|  \|  \|  \|  \| \| **Pancreatic cancer cases with other single KRAS cfDNA mutation reported in COSMIC** \| \| \|  \|  \|  \|  \|  \|  \|  \|  \|  \|  \|  \|  \| \|  \| Status \| Sample ID \| Stage \| QVAL^a^ \| Read Depth \| Mutated Reads \| RVSB^b^ \|  \| HGVS cDNA \| HGVS protein \| Mutation type \| AF^c^ (%) \|  \| comments \| \|  \| Unknown/Missing \| CA82 \| Regional \| 70.36 \| 12324 \| 20 \| 0.63 \|  \| c.176C>A \| p.A59E \| missense \| 0.16 \|  \| NA \| \|  \| Endocrine \| CA83 \| Systemic \| 34.36 \| 6035 \| 13 \| 0.63 \|  \| c.176C>G \| p.A59G \| missense \| 0.22 \|  \| NA \| \|  \| Unknown/Missing \| CA84 \| Regional \| 31.29 \| 2981 \| 7 \| 1.00 \|  \| c.A209C \| p.Q70P \| missense \| 0.23 \|  \| NA \| \|  \| Unknown/Missing \| CA85 \| Regional \| 242.26 \| 5310 \| 19 \| 0.58 \|  \| c.190T>G \| p.Y64D \| missense \| 0.36 \|  \| NA \| \|  \| PDAC \| CA86 \| Local \| 36.22 \| 2188 \| 8 \| 0.53 \|  \| c.199A>C \| p.M67L \| missense \| 0.37 \|  \| NA \| \|  \| PDAC \| CA87 \| Regional \| 32.98 \| 2404 \| 9 \| 0.78 \|  \| c.182A>C \| p.Q61P \| missense \| 0.37 \|  \| NA \| \|  \| Endocrine \| CA88 \| Regional \| 241.03 \| 4047 \| 59 \| 0.50 \|  \| c.179G>A \| p.G60D \| missense \| 1.46 \|  \| NA \| \|  \| PDAC \| CA89 \| Systemic \| 45.28 \| 2680 \| 44 \| 0.62 \|  \| c.A214G \| p.M72V \| missense \| 1.64 \|  \| NA \| \|  \| PDAC \| CA90 \| Regional \| Infinite \| 9110 \| 4225 \| 0.51 \|  \| c.24A>G \| p.V8V \| silent \| 46.38 \|  \| NA \| \|  \|  \|  \|  \|  \|  \|  \|  \|  \|  \|  \|  \|  \|  \|  \| \| **Pancreatic cancer cases with multiple KRAS cfDNA mutations reported in COSMIC** \| \| \| \| \| \| \|  \|  \|  \|  \|  \|  \|  \|  \| \|  \| Status \| Sample ID \| Stage \| QVAL^a^ \| Read Depth \| Mutated Reads \| RVSB^b^ \|  \| HGVS cDNA \| HGVS protein \| Mutation type \| AF^c^ (%) \|  \| comments \| \|  \| Unknown/Missing \| CA91 \| Unknown \| 34.19 \| 4335 \| 28 \| 0.54 \|  \| c.35G>A \| p.G12D \| missense \| 0.65 \|  \| not measurable \| \|  \| Unknown/Missing \| CA91 \| Unknown \| 35.60 \| 3461 \| 7 \| 0.50 \|  \| c.186G>T \| p.E62D \| missense \| 0.20 \|  \| not measurable \| \|  \| PDAC \| CA92 \| Local \| 59.76 \| 6069 \| 6 \| 0.53 \|  \| c.37G>C \| p.G13R \| missense \| 0.10 \|  \| mutations on different reads \| \|  \| PDAC \| CA92 \| Local \| 1973.81 \| 6093 \| 698 \| 0.58 \|  \| c.24A>G \| p.V8V \| silent \| 11.46 \|  \| mutations on different reads \| \|  \| Unknown/Missing \| CA93 \| Unknown \| 31.53 \| 5151 \| 4 \| 0.60 \|  \| c.37G>C \| p.G13R \| missense \| 0.08 \|  \| mutations on different reads \| \|  \| Unknown/Missing \| CA93 \| Unknown \| Infinite \| 5158 \| 1332 \| 0.56 \|  \| c.35G>T \| p.G12V \| missense \| 25.82 \|  \| mutations on different reads \| \|  \|  \|  \|  \|  \|  \|  \|  \|  \|  \|  \|  \|  \|  \|  \| \| **Healthy controls with a single KRAS cfDNA mutation at hotspot codon 12, 13 or 61 reported in pancreas** \| \| \| \| \| \| \| \| \| \|  \|  \|  \|  \|  \| \|  \| Status \| Sample ID \| Stage \| QVAL^a^ \| Read Depth \| Mutated Reads \| RVSB^b^ \|  \| HGVS cDNA \| HGVS protein \| Mutation type \| AF^c^ (%) \|  \| comments \| \|  \| Healthy control \| CO01 \| NA \| 43.18 \| 6450 \| 13 \| 0.62 \|  \| c.181C>A \| p.Q61K \| missense \| 0.20 \|  \| NA \| \|  \| Healthy control \| CO02 \| NA \| 45.94 \| 2214 \| 5 \| 0.53 \|  \| c.34G>C \| p.G12R \| missense \| 0.23 \|  \| NA \| \|  \| Healthy control \| CO03 \| NA \| 116.88 \| 3794 \| 12 \| 0.52 \|  \| c.34G>C \| p.G12R \| missense \| 0.32 \|  \| NA \| \|  \| Healthy control \| CO04 \| NA \| 45.18 \| 5564 \| 39 \| 0.63 \|  \| c.35G>A \| p.G12D \| missense \| 0.70 \|  \| NA \| \|  \| Healthy control \| CO05 \| NA \| 87.30 \| 3040 \| 37 \| 0.90 \|  \| c.35G>A \| p.G12D \| missense \| 1.22 \|  \| NA \| \|  \| Healthy control \| CO06 \| NA \| 951.05 \| 7119 \| 250 \| 0.52 \|  \| c.183A>T \| p.Q61H \| missense \| 3.51 \|  \| NA \| \|  \|  \|  \|  \|  \|  \|  \|  \|  \|  \|  \|  \|  \|  \|  \| \| **Healthy controls with other single KRAS cfDNA mutation reported in COSMIC** \| \| \| \| \| \| \|  \|  \|  \|  \|  \|  \|  \|  \| \|  \| Status \| Sample ID \| Stage \| QVAL^a^ \| Read Depth \| Mutated Reads \| RVSB^b^ \|  \| HGVS cDNA \| HGVS protein \| Mutation type \| AF^c^ (%) \|  \| comments \| \|  \| Healthy control \| CO07 \| NA \| 36.52 \| 4794 \| 5 \| 0.63 \|  \| c.181C>G \| p.Q61E \| missense \| 0.10 \|  \| NA \| \|  \| Healthy control \| CO08 \| NA \| 48.30 \| 7938 \| 12 \| 0.65 \|  \| c.45C>A \| p.G15G \| silent \| 0.15 \|  \| NA \| \|  \| Healthy control \| CO09 \| NA \| 30.08 \| 3027 \| 5 \| 1.00 \|  \| c.45C>G \| p.G15G \| silent \| 0.17 \|  \| NA \| \|  \| Healthy control \| CO10 \| NA \| 38.35 \| 4141 \| 8 \| 0.70 \|  \| c.186G>T \| p.E62D \| missense \| 0.19 \|  \| NA \| \|  \| Healthy control \| CO11 \| NA \| 68.18 \| 4163 \| 9 \| 0.58 \|  \| c.204G>C \| p.R68S \| missense \| 0.22 \|  \| NA \| \|  \| Healthy control \| CO12 \| NA \| Infinite \| 2299 \| 1080 \| 0.50 \|  \| c.24A>G \| p.V8V \| silent \| 46.98 \|  \| NA \| \|  \|  \|  \|  \|  \|  \|  \|  \|  \|  \|  \|  \|  \|  \|  \| \| **Healthy controls with multiple KRAS cfDNA mutations reported in COSMIC** \| \| \| \| \| \| \|  \|  \|  \|  \|  \|  \|  \|  \| \|  \| Status \| Sample ID \| Stage \| QVAL^a^ \| Read Depth \| Mutated Reads \| RVSB^b^ \|  \| HGVS cDNA \| HGVS protein \| Mutation type \| AF^c^ (%) \|  \| comments \| \|  \| Healthy control \| CO13 \| NA \| 57.22 \| 5579 \| 8 \| 0.54 \|  \| c.34G>C \| p.G12R \| missense \| 0.14 \|  \| mutations on different reads \| \|  \| Healthy control \| CO13 \| NA \| 39.85 \| 5812 \| 15 \| 0.54 \|  \| c.35G>T \| p.G12V \| missense \| 0.26 \|  \| mutations on different reads \| \|  \| Healthy control \| CO14 \| NA \| 71.88 \| 6515 \| 10 \| 0.68 \|  \| c.34G>C \| p.G12R \| missense \| 0.15 \|  \| mutations on different reads \| \|  \| Healthy control \| CO14 \| NA \| 85.76 \| 6734 \| 31 \| 0.71 \|  \| c.35G>T \| p.G12V \| missense \| 0.46 \|  \| mutations on different reads \| \|  \| Healthy control \| CO14 \| NA \| 50.15 \| 6734 \| 48 \| 0.57 \|  \| c.35G>A \| p.G12D \| missense \| 0.71 \|  \| mutations on different reads \| \|  \| Healthy control \| CO15 \| NA \| 43.80 \| 2980 \| 23 \| 0.52 \|  \| c.182A>T \| p.Q61L \| missense \| 0.77 \|  \| mutations on different reads \| \|  \| healthy control \| CO15 \| NA \| 426.17 \| 3148 \| 706 \| 0.53 \|  \| c.175G>A \| p.A59T \| missense \| 22.43 \|  \| mutations on different reads \| \|  \|  \|  \|  \|  \|  \|  \|  \|  \|  \|  \|  \|  \|  \|  \| \| **Chronic pancreatitis cases with a single KRAS cfDNA mutation at hotspot codon 12, 13 or 61 reported in pancreas** \| \| \| \| \| \| \| \| \| \|  \|  \|  \|  \|  \| \|  \| Status \| Sample ID \| Stage \| QVAL^a^ \| Read Depth \| Mutated Reads \| RVSB^b^ \|  \| HGVS cDNA \| HGVS protein \| Mutation type \| AF^c^ (%) \|  \| comments \| \|  \| Chronic pancreatitis \| CH01 \| NA \| 61.75 \| 9849 \| 20 \| 0.56 \|  \| c.34G>T \| p.G12C \| missense \| 0.20 \|  \| NA \| \|  \| Chronic pancreatitis \| CH02 \| NA \| Infinite \| 5570 \| 934 \| 0.62 \|  \| c.34G>T \| p.G12C \| missense \| 16.77 \|  \| NA \| \|  \|  \|  \|  \|  \|  \|  \|  \|  \|  \|  \|  \|  \|  \|  \| \| **Chronic pancreatitis cases with other single KRAS cfDNA mutation reported in COSMIC** \| \| \| \| \| \| \|  \|  \|  \|  \|  \|  \|  \|  \| \|  \| Status \| Sample ID \| Stage \| QVAL^a^ \| Read Depth \| Mutated Reads \| RVSB^b^ \|  \| HGVS cDNA \| HGVS protein \| Mutation type \| AF^c^ (%) \|  \| comments \| \|  \| Chronic pancreatitis \| CH03 \| NA \| 51.36 \| 5649 \| 11 \| 0.56 \|  \| c.45C>A \| p.G15G \| silent \| 0.19 \|  \| NA \| \|  \| Chronic pancreatitis \| CH04 \| NA \| 31.25 \| 3863 \| 19 \| 0.65 \|  \| c.187G>A \| p.E63K \| missense \| 0.49 \|  \| NA \| \|  \|  \|  \|  \|  \|  \|  \|  \|  \|  \|  \|  \|  \|  \|  \| \| **Chronic pancreatitis cases with multiple KRAS cfDNA mutations reported in COSMIC** \| \| \| \| \| \| \|  \|  \|  \|  \|  \|  \|  \|  \| \|  \| Status \| Sample ID \| Stage \| QVAL^a^ \| Read Depth \| Mutated Reads \| RVSB^b^ \|  \| HGVS cDNA \| HGVS protein \| Mutation type \| AF^c^ (%) \|  \| comments \| \|  \| Chronic pancreatitis \| CH05 \| NA \| 47.37 \| 3632 \| 6 \| 0.62 \|  \| c.34G>C \| p.G12R \| missense \| 0.17 \|  \| mutations on different reads \| \|  \| Chronic pancreatitis \| CH05 \| NA \| 46.73 \| 3837 \| 13 \| 0.77 \|  \| c.35G>T \| p.G12V \| missense \| 0.34 \|  \| mutations on different reads \| \|  \| Chronic pancreatitis \| CH06 \| NA \| 97.46 \| 4484 \| 11 \| 0.54 \|  \| c.34G>C \| p.G12R \| missense \| 0.25 \|  \| mutations on different reads \| \|  \| Chronic pancreatitis \| CH06 \| NA \| 48.27 \| 4623 \| 15 \| 0.67 \|  \| c.35G>T \| p.G12V \| missense \| 0.32 \|  \| mutations on different reads \| \|  \| Chronic pancreatitis \| CH06 \| NA \| 58.43 \| 4623 \| 39 \| 0.51 \|  \| c.35G>A \| p.G12D \| missense \| 0.84 \|  \| mutations on different reads \| \| \| --- \| --- \| --- \| --- \| --- \| --- \| --- \| --- \| --- \| --- \| --- \| --- \| --- \| --- \| --- \| --- \| --- \| --- \| --- \| --- \| --- \| --- \| --- \| --- \| --- \| --- \| --- \| --- \| --- \| --- \| --- \| --- \| --- \| --- \| --- \| --- \| --- \| --- \| --- \| --- \| --- \| --- \| --- \| --- \| --- \| --- \| --- \| --- \| --- \| --- \| --- \| --- \| --- \| --- \| --- \| --- \| --- \| --- \| --- \| --- \| --- \| --- \| --- \| --- \| --- \| --- \| --- \| --- \| --- \| --- \| --- \| --- \| --- \| --- \| --- \| --- \| --- \| --- \| --- \| --- \| --- \| --- \| --- \| --- \| --- \| --- \| --- \| --- \| --- \| --- \| --- \| --- \| --- \| --- \| --- \| --- \| --- \| --- \| --- \| --- \| --- \| --- \| --- \| --- \| --- \| --- \| --- \| --- \| --- \| --- \| --- \| --- \| --- \| --- \| --- \| --- \| --- \| --- \| --- \| --- \| --- \| --- \| --- \| --- \| --- \| --- \| --- \| --- \| --- \| --- \| --- \| --- \| --- \| --- \| --- \| --- \| --- \| --- \| --- \| --- \| --- \| --- \| --- \| --- \| --- \| --- \| --- \| --- \| --- \| --- \| --- \| --- \| --- \| --- \| --- \| --- \| --- \| --- \| --- \| --- \| --- \| --- \| --- \| --- \| --- \| --- \| --- \| --- \| --- \| --- \| --- \| --- \| --- \| --- \| --- \| --- \| --- \| --- \| --- \| --- \| --- \| --- \| --- \| --- \| --- \| --- \| --- \| --- \| --- \| --- \| --- \| --- \| --- \| --- \| --- \| --- \| --- \| --- \| --- \| --- \| --- \| --- \| --- \| --- \| --- \| --- \| --- \| --- \| --- \| --- \| --- \| --- \| --- \| --- \| --- \| --- \| --- \| --- \| --- \| --- \| --- \| --- \| --- \| --- \| --- \| --- \| --- \| --- \| --- \| --- \| --- \| --- \| --- \| --- \| --- \| --- \| --- \| --- \| --- \| --- \| --- \| --- \| --- \| --- \| --- \| --- \| --- \| --- \| --- \| --- \| --- \| --- \| --- \| --- \| --- \| --- \| --- \| --- \| --- \| --- \| --- \| --- \| --- \| --- \| --- \| --- \| --- \| --- \| --- \| --- \| --- \| --- \| --- \| --- \| --- \| --- \| --- \| --- \| --- \| --- \| --- \| --- \| --- \| --- \| --- \| --- \| --- \| --- \| --- \| --- \| --- \| --- \| --- \| --- \| --- \| --- \| --- \| --- \| --- \| --- \| --- \| --- \| --- \| --- \| --- \| --- \| --- \| --- \| --- \| --- \| --- \| --- \| --- \| --- \| --- \| --- \| --- \| --- \| --- \| --- \| --- \| --- \| --- \| --- \| --- \| --- \| --- \| --- \| --- \| --- \| --- \| --- \| --- \| --- \| --- \| --- \| --- \| --- \| --- \| --- \| --- \| --- \| --- \| --- \| --- \| --- \| --- \| --- \| --- \| --- \| --- \| --- \| --- \| --- \| --- \| --- \| --- \| --- \| --- \| --- \| --- \| --- \| --- \| --- \| --- \| --- \| --- \| --- \| --- \| --- \| --- \| --- \| --- \| --- \| --- \| --- \| --- \| --- \| --- \| --- \| --- \| --- \| --- \| --- \| --- \| --- \| --- \| --- \| --- \| --- \| --- \| --- \| --- \| --- \| --- \| --- \| --- \| --- \| --- \| --- \| --- \| --- \| --- \| --- \| --- \| --- \| --- \| --- \| --- \| --- \| --- \| --- \| --- \| --- \| --- \| --- \| --- \| --- \| --- \| --- \| --- \| --- \| --- \| --- \| --- \| --- \| --- \| --- \| --- \| --- \| --- \| --- \| --- \| --- \| --- \| --- \| --- \| --- \| --- \| --- \| --- \| --- \| --- \| --- \| --- \| --- \| --- \| --- \| --- \| --- \| --- \| --- \| --- \| --- \| --- \| --- \| --- \| --- \| --- \| --- \| --- \| --- \| --- \| --- \| --- \| --- \| --- \| --- \| --- \| --- \| --- \| --- \| --- \| --- \| --- \| --- \| --- \| --- \| --- \| --- \| --- \| --- \| --- \| --- \| --- \| --- \| --- \| --- \| --- \| --- \| --- \| --- \| --- \| --- \| --- \| --- \| --- \| --- \| --- \| --- \| --- \| --- \| --- \| --- \| --- \| --- \| --- \| --- \| --- \| --- \| --- \| --- \| --- \| --- \| --- \| --- \| --- \| --- \| --- \| --- \| --- \| --- \| --- \| --- \| --- \| --- \| --- \| --- \| --- \| --- \| --- \| --- \| --- \| --- \| --- \| --- \| --- \| --- \| --- \| --- \| --- \| --- \| --- \| --- \| --- \| --- \| --- \| --- \| --- \| --- \| --- \| --- \| --- \| --- \| --- \| --- \| --- \| --- \| --- \| --- \| --- \| --- \| --- \| --- \| --- \| --- \| --- \| --- \| --- \| --- \| --- \| --- \| --- \| --- \| --- \| --- \| --- \| --- \| --- \| --- \| --- \| --- \| --- \| --- \| --- \| --- \| --- \| --- \| --- \| --- \| --- \| --- \| --- \| --- \| --- \| --- \| --- \| --- \| --- \| --- \| --- \| --- \| --- \| --- \| --- \| --- \| --- \| --- \| --- \| --- \| --- \| --- \| --- \| --- \| --- \| --- \| --- \| --- \| --- \| --- \| --- \| --- \| --- \| --- \| --- \| --- \| --- \| --- \| --- \| --- \| --- \| --- \| --- \| --- \| --- \| --- \| --- \| --- \| --- \| --- \| --- \| --- \| --- \| --- \| --- \| --- \| --- \| --- \| --- \| --- \| --- \| --- \| --- \| --- \| --- \| --- \| --- \| --- \| --- \| --- \| --- \| --- \| --- \| --- \| --- \| --- \| --- \| --- \| --- \| --- \| --- \| --- \| --- \| --- \| --- \| --- \| --- \| --- \| --- \| --- \| --- \| --- \| --- \| --- \| --- \| --- \| --- \| --- \| --- \| --- \| --- \| --- \| --- \| --- \| --- \| --- \| --- \| --- \| --- \| --- \| --- \| --- \| --- \| --- \| --- \| --- \| --- \| --- \| --- \| --- \| --- \| --- \| --- \| --- \| --- \| --- \| --- \| --- \| --- \| --- \| --- \| --- \| --- \| --- \| --- \| --- \| --- \| --- \| --- \| --- \| --- \| --- \| --- \| --- \| --- \| --- \| --- \| --- \| --- \| --- \| --- \| --- \| --- \| --- \| --- \| --- \| --- \| --- \| --- \| --- \| --- \| --- \| --- \| --- \| --- \| --- \| --- \| --- \| --- \| --- \| --- \| --- \| --- \| --- \| --- \| --- \| --- \| --- \| --- \| --- \| --- \| --- \| --- \| --- \| --- \| --- \| --- \| --- \| --- \| --- \| --- \| --- \| --- \| --- \| --- \| --- \| --- \| --- \| --- \| --- \| --- \| --- \| --- \| --- \| --- \| --- \| --- \| --- \| --- \| --- \| --- \| --- \| --- \| --- \| --- \| --- \| --- \| --- \| --- \| --- \| --- \| --- \| --- \| --- \| --- \| --- \| --- \| --- \| --- \| --- \| --- \| --- \| --- \| --- \| --- \| --- \| --- \| --- \| --- \| --- \| --- \| --- \| --- \| --- \| --- \| --- \| --- \| --- \| --- \| --- \| --- \| --- \| --- \| --- \| --- \| --- \| --- \| --- \| --- \| --- \| --- \| --- \| --- \| --- \| --- \| --- \| --- \| --- \| --- \| --- \| --- \| --- \| --- \| --- \| --- \| --- \| --- \| --- \| --- \| --- \| --- \| --- \| --- \| --- \| --- \| --- \| --- \| --- \| --- \| --- \| --- \| --- \| --- \| --- \| --- \| --- \| --- \| --- \| --- \| --- \| --- \| --- \| --- \| --- \| --- \| --- \| --- \| --- \| --- \| --- \| --- \| --- \| --- \| --- \| --- \| --- \| --- \| --- \| --- \| --- \| --- \| --- \| --- \| --- \| --- \| --- \| --- \| --- \| --- \| --- \| --- \| --- \| --- \| --- \| --- \| --- \| --- \| --- \| --- \| --- \| --- \| --- \| --- \| --- \| --- \| --- \| --- \| --- \| --- \| --- \| --- \| --- \| --- \| --- \| --- \| --- \| --- \| --- \| --- \| --- \| --- \| --- \| --- \| --- \| --- \| --- \| --- \| --- \| --- \| --- \| --- \| --- \| --- \| --- \| --- \| --- \| --- \| --- \| --- \| --- \| --- \| --- \| --- \| --- \| --- \| --- \| --- \| --- \| --- \| --- \| --- \| --- \| --- \| --- \| --- \| --- \| --- \| --- \| --- \| --- \| --- \| --- \| --- \| --- \| --- \| --- \| --- \| --- \| --- \| --- \| --- \| --- \| --- \| --- \| --- \| --- \| --- \| --- \| --- \| --- \| --- \| --- \| --- \| --- \| --- \| --- \| --- \| --- \| --- \| --- \| --- \| --- \| --- \| --- \| --- \| --- \| --- \| --- \| --- \| --- \| --- \| --- \| --- \| --- \| --- \| --- \| --- \| --- \| --- \| --- \| --- \| --- \| --- \| --- \| --- \| --- \| --- \| --- \| --- \| --- \| --- \| --- \| --- \| --- \| --- \| --- \| --- \| --- \| --- \| --- \| --- \| --- \| --- \| --- \| --- \| --- \| --- \| --- \| --- \| --- \| --- \| --- \| --- \| --- \| --- \| --- \| --- \| --- \| --- \| --- \| --- \| --- \| --- \| --- \| --- \| --- \| --- \| --- \| --- \| --- \| --- \| --- \| --- \| --- \| --- \| --- \| --- \| --- \| --- \| --- \| --- \| --- \| --- \| --- \| --- \| --- \| --- \| --- \| --- \| --- \| --- \| --- \| --- \| --- \| --- \| --- \| --- \| --- \| --- \| --- \| --- \| --- \| --- \| --- \| --- \| --- \| --- \| --- \| --- \| --- \| --- \| --- \| --- \| --- \| --- \| --- \| --- \| --- \| --- \| --- \| --- \| --- \| --- \| --- \| --- \| --- \| --- \| --- \| --- \| --- \| --- \| --- \| --- \| --- \| --- \| --- \| --- \| --- \| --- \| --- \| --- \| --- \| --- \| --- \| --- \| --- \| --- \| --- \| --- \| --- \| --- \| --- \| --- \| --- \| --- \| --- \| --- \| --- \| --- \| --- \| --- \| --- \| --- \| --- \| --- \| --- \| --- \| --- \| --- \| --- \| --- \| --- \| --- \| --- \| --- \| --- \| --- \| --- \| --- \| --- \| --- \| --- \| --- \| --- \| --- \| --- \| --- \| --- \| --- \| --- \| --- \| --- \| --- \| --- \| --- \| --- \| --- \| --- \| --- \| --- \| --- \| --- \| --- \| --- \| --- \| --- \| --- \| --- \| --- \| --- \| --- \| --- \| --- \| --- \| --- \| --- \| --- \| --- \| --- \| --- \| --- \| --- \| --- \| --- \| --- \| --- \| --- \| --- \| --- \| --- \| --- \| --- \| --- \| --- \| --- \| --- \| --- \| --- \| --- \| --- \| --- \| --- \| --- \| --- \| --- \| --- \| --- \| --- \| --- \| --- \| --- \| --- \| --- \| --- \| --- \| --- \| --- \| --- \| --- \| --- \| --- \| --- \| --- \| --- \| --- \| --- \| --- \| --- \| --- \| --- \| --- \| --- \| --- \| --- \| --- \| --- \| --- \| --- \| --- \| --- \| --- \| --- \| --- \| --- \| --- \| --- \| --- \| --- \| --- \| --- \| --- \| --- \| --- \| --- \| --- \| --- \| --- \| --- \| --- \| --- \| --- \| --- \| --- \| --- \| --- \| --- \| --- \| --- \| --- \| --- \| --- \| --- \| --- \| --- \| --- \| --- \| --- \| --- \| --- \| --- \| --- \| --- \| --- \| --- \| --- \| --- \| --- \| --- \| --- \| --- \| --- \| --- \| --- \| --- \| --- \| --- \| --- \| --- \| --- \| --- \| --- \| --- \| --- \| --- \| --- \| --- \| --- \| --- \| --- \| --- \| --- \| --- \| --- \| --- \| --- \| --- \| --- \| --- \| --- \| --- \| --- \| --- \| --- \| --- \| --- \| --- \| --- \| --- \| --- \| --- \| --- \| --- \| --- \| --- \| --- \| --- \| --- \| --- \| --- \| --- \| --- \| --- \| --- \| --- \| --- \| --- \| --- \| --- \| --- \| --- \| --- \| --- \| --- \| --- \| --- \| --- \| --- \| --- \| --- \| --- \| --- \| --- \| --- \| --- \| --- \| --- \| --- \| --- \| --- \| --- \| --- \| --- \| --- \| --- \| --- \| --- \| --- \| --- \| --- \| --- \| --- \| --- \| --- \| --- \| --- \| --- \| --- \| --- \| --- \| --- \| --- \| --- \| --- \| --- \| --- \| --- \| --- \| --- \| --- \| --- \| --- \| --- \| --- \| --- \| --- \| --- \| --- \| --- \| --- \| --- \| --- \| --- \| --- \| --- \| --- \| --- \| --- \| --- \| --- \| --- \| --- \| --- \| --- \| --- \| --- \| --- \| --- \| --- \| --- \| --- \| --- \| --- \| --- \| --- \| --- \| --- \| --- \| --- \| --- \| --- \| --- \| --- \| --- \| --- \| --- \| --- \| --- \| --- \| --- \| --- \| --- \| --- \| --- \| --- \| --- \| --- \| --- \| --- \| --- \| --- \| --- \| --- \| --- \| --- \| --- \| --- \| --- \| --- \| --- \| --- \| --- \| --- \| --- \| --- \| --- \| --- \| --- \| --- \| --- \| --- \| --- \| --- \| --- \| --- \| --- \| --- \| --- \| --- \| --- \| --- \| --- \| --- \| --- \| --- \| --- \| --- \| --- \| --- \| --- \| --- \| --- \| --- \| --- \| --- \| --- \| --- \| --- \| --- \| --- \| --- \| --- \| --- \| --- \| --- \| --- \| --- \| --- \| --- \| --- \| --- \| --- \| --- \| --- \| --- \| --- \| --- \| --- \| --- \| --- \| --- \| --- \| --- \| --- \| --- \| --- \| --- \| --- \| --- \| --- \| --- \| --- \| --- \| --- \| --- \| --- \| --- \| --- \| --- \| --- \| --- \| --- \| --- \| --- \| --- \| --- \| --- \| --- \| --- \| --- \| --- \| --- \| --- \| --- \| --- \| --- \| --- \| --- \| --- \| --- \| --- \| --- \| --- \| --- \| --- \| --- \| --- \| --- \| --- \| --- \| --- \| --- \| --- \| --- \| --- \| --- \| --- \| --- \| --- \| --- \| --- \| --- \| --- \| --- \| --- \| --- \| --- \| --- \| --- \| --- \| --- \| --- \| --- \| --- \| --- \| --- \| --- \| --- \| --- \| --- \| --- \| --- \| --- \| --- \| --- \| --- \| --- \| --- \| --- \| --- \| --- \| --- \| --- \| --- \| --- \| --- \| --- \| --- \| --- \| --- \| --- \| --- \| --- \| --- \| --- \| --- \| --- \| --- \| --- \| --- \| --- \| --- \| --- \| --- \| --- \| --- \| --- \| --- \| --- \| --- \| --- \| --- \| --- \| --- \| --- \| --- \| --- \| --- \| --- \| --- \| --- \| --- \| --- \| --- \| --- \| --- \| --- \| --- \| --- \| --- \| --- \| --- \| --- \| --- \| --- \| --- \| --- \| --- \| --- \| --- \| --- \| --- \| --- \| --- \| --- \| --- \| --- \| --- \| --- \| --- \| --- \| --- \| --- \| --- \| --- \| --- \| --- \| --- \| --- \| --- \| --- \| --- \| --- \| --- \| --- \| --- \| --- \| --- \| --- \| --- \| --- \| --- \| --- \| --- \| --- \| --- \| --- \| --- \| --- \| --- \| --- \| --- \| --- \| --- \| --- \| --- \| --- \| --- \| --- \| --- \| --- \| --- \| --- \| --- \| --- \| --- \| --- \| --- \| --- \| --- \| --- \| --- \| --- \| --- \| --- \| --- \| --- \| --- \| --- \| --- \| --- \| --- \| --- \| --- \| --- \| --- \| --- \| --- \| --- \| --- \| --- \| --- \| --- \| --- \| --- \| --- \| --- \| --- \| --- \| --- \| --- \| --- \| --- \| --- \| --- \| --- \| --- \| --- \| --- \| --- \| --- \| --- \| --- \| --- \| --- \| --- \| --- \| --- \| --- \| --- \| --- \| --- \| --- \| --- \| --- \| --- \| --- \| --- \| --- \| --- \| --- \| --- \| --- \| --- \| --- \| --- \| --- \| --- \| --- \| --- \| --- \| --- \| --- \| --- \| --- \| --- \| --- \| --- \| --- \| --- \| --- \| --- \| --- \| --- \| --- \| --- \| --- \| --- \| --- \| --- \| --- \| --- \| --- \| --- \| --- \| --- \| --- \| --- \| --- \| --- \| --- \| --- \| --- \| --- \| --- \| --- \| --- \| --- \| --- \| --- \| --- \| --- \| --- \| --- \| --- \| --- \| --- \| --- \| --- \| --- \| --- \| --- \| --- \| --- \| --- \| --- \| --- \| --- \| --- \| --- \| --- \| --- \| --- \| --- \| --- \| --- \| --- \| --- \| --- \| --- \| --- \| --- \| --- \| --- \| --- \| --- \| --- \| --- \| --- \| --- \| --- \| --- \| --- \| --- \| --- \| --- \| --- \| --- \| --- \| --- \| --- \| --- \| --- \| --- \| --- \| --- \| --- \| --- \| --- \| --- \| --- \| --- \| --- \| --- \| --- \| --- \| --- \| --- \| --- \| --- \| --- \| --- \| --- \| --- \| --- \| --- \| --- \| --- \| --- \| --- \| --- \| --- \| --- \| --- \| --- \| --- \| --- \| --- \| --- \| --- \| --- \| --- \| --- \| --- \| --- \| --- \| --- \| --- \| --- \| --- \| --- \| --- \| --- \| --- \| --- \| --- \| --- \| --- \| --- \| --- \| --- \| --- \| --- \| --- \| --- \| --- \| --- \| --- \| --- \| --- \| --- \| --- \| --- \| --- \| --- \| --- \| --- \| --- \| --- \| --- \| --- \| --- \| --- \| --- \| --- \| --- \| --- \| --- \| --- \| --- \| --- \| --- \| --- \| --- \| --- \| --- \| --- \| --- \| --- \| --- \| --- \| --- \| --- \| --- \| --- \| --- \| --- \| --- \| --- \| --- \| --- \| --- \| --- \| --- \| --- \| --- \| --- \| --- \| --- \| --- \| --- \| --- \| --- \| --- \| --- \| --- \| --- \| --- \| --- \| --- \| --- \| --- \| --- \| --- \| --- \| --- \| --- \| --- \| --- \| --- \| --- \| --- \| --- \| --- \| --- \| --- \| --- \| --- \| --- \| --- \| --- \| --- \| --- \| --- \| --- \| --- \| --- \| |
| --- | --- | --- | --- | --- | --- | --- | --- | --- | --- | --- | --- | --- | --- | --- | --- | --- | --- | --- | --- | --- | --- | --- | --- | --- | --- | --- | --- | --- | --- | --- | --- | --- | --- | --- | --- | --- | --- | --- | --- | --- | --- | --- | --- | --- | --- | --- | --- | --- | --- | --- | --- | --- | --- | --- | --- | --- | --- | --- | --- | --- | --- | --- | --- | --- | --- | --- | --- | --- | --- | --- | --- | --- | --- | --- | --- | --- | --- | --- | --- | --- | --- | --- | --- | --- | --- | --- | --- | --- | --- | --- | --- | --- | --- | --- | --- | --- | --- | --- | --- | --- | --- | --- | --- | --- | --- | --- | --- | --- | --- | --- | --- | --- | --- | --- | --- | --- | --- | --- | --- | --- | --- | --- | --- | --- | --- | --- | --- | --- | --- | --- | --- | --- | --- | --- | --- | --- | --- | --- | --- | --- | --- | --- | --- | --- | --- | --- | --- | --- | --- | --- | --- | --- | --- | --- | --- | --- | --- | --- | --- | --- | --- | --- | --- | --- | --- | --- | --- | --- | --- | --- | --- | --- | --- | --- | --- | --- | --- | --- | --- | --- | --- | --- | --- | --- | --- | --- | --- | --- | --- | --- | --- | --- | --- | --- | --- | --- | --- | --- | --- | --- | --- | --- | --- | --- | --- | --- | --- | --- | --- | --- | --- | --- | --- | --- | --- | --- | --- | --- | --- | --- | --- | --- | --- | --- | --- | --- | --- | --- | --- | --- | --- | --- | --- | --- | --- | --- | --- | --- | --- | --- | --- | --- | --- | --- | --- | --- | --- | --- | --- | --- | --- | --- | --- | --- | --- | --- | --- | --- | --- | --- | --- | --- | --- | --- | --- | --- | --- | --- | --- | --- | --- | --- | --- | --- | --- | --- | --- | --- | --- | --- | --- | --- | --- | --- | --- | --- | --- | --- | --- | --- | --- | --- | --- | --- | --- | --- | --- | --- | --- | --- | --- | --- | --- | --- | --- | --- | --- | --- | --- | --- | --- | --- | --- | --- | --- | --- | --- | --- | --- | --- | --- | --- | --- | --- | --- | --- | --- | --- | --- | --- | --- | --- | --- | --- | --- | --- | --- | --- | --- | --- | --- | --- | --- | --- | --- | --- | --- | --- | --- | --- | --- | --- | --- | --- | --- | --- | --- | --- | --- | --- | --- | --- | --- | --- | --- | --- | --- | --- | --- | --- | --- | --- | --- | --- | --- | --- | --- | --- | --- | --- | --- | --- | --- | --- | --- | --- | --- | --- | --- | --- | --- | --- | --- | --- | --- | --- | --- | --- | --- | --- | --- | --- | --- | --- | --- | --- | --- | --- | --- | --- | --- | --- | --- | --- | --- | --- | --- | --- | --- | --- | --- | --- | --- | --- | --- | --- | --- | --- | --- | --- | --- | --- | --- | --- | --- | --- | --- | --- | --- | --- | --- | --- | --- | --- | --- | --- | --- | --- | --- | --- | --- | --- | --- | --- | --- | --- | --- | --- | --- | --- | --- | --- | --- | --- | --- | --- | --- | --- | --- | --- | --- | --- | --- | --- | --- | --- | --- | --- | --- | --- | --- | --- | --- | --- | --- | --- | --- | --- | --- | --- | --- | --- | --- | --- | --- | --- | --- | --- | --- | --- | --- | --- | --- | --- | --- | --- | --- | --- | --- | --- | --- | --- | --- | --- | --- | --- | --- | --- | --- | --- | --- | --- | --- | --- | --- | --- | --- | --- | --- | --- | --- | --- | --- | --- | --- | --- | --- | --- | --- | --- | --- | --- | --- | --- | --- | --- | --- | --- | --- | --- | --- | --- | --- | --- | --- | --- | --- | --- | --- | --- | --- | --- | --- | --- | --- | --- | --- | --- | --- | --- | --- | --- | --- | --- | --- | --- | --- | --- | --- | --- | --- | --- | --- | --- | --- | --- | --- | --- | --- | --- | --- | --- | --- | --- | --- | --- | --- | --- | --- | --- | --- | --- | --- | --- | --- | --- | --- | --- | --- | --- | --- | --- | --- | --- | --- | --- | --- | --- | --- | --- | --- | --- | --- | --- | --- | --- | --- | --- | --- | --- | --- | --- | --- | --- | --- | --- | --- | --- | --- | --- | --- | --- | --- | --- | --- | --- | --- | --- | --- | --- | --- | --- | --- | --- | --- | --- | --- | --- | --- | --- | --- | --- | --- | --- | --- | --- | --- | --- | --- | --- | --- | --- | --- | --- | --- | --- | --- | --- | --- | --- | --- | --- | --- | --- | --- | --- | --- | --- | --- | --- | --- | --- | --- | --- | --- | --- | --- | --- | --- | --- | --- | --- | --- | --- | --- | --- | --- | --- | --- | --- | --- | --- | --- | --- | --- | --- | --- | --- | --- | --- | --- | --- | --- | --- | --- | --- | --- | --- | --- | --- | --- | --- | --- | --- | --- | --- | --- | --- | --- | --- | --- | --- | --- | --- | --- | --- | --- | --- | --- | --- | --- | --- | --- | --- | --- | --- | --- | --- | --- | --- | --- | --- | --- | --- | --- | --- | --- | --- | --- | --- | --- | --- | --- | --- | --- | --- | --- | --- | --- | --- | --- | --- | --- | --- | --- | --- | --- | --- | --- | --- | --- | --- | --- | --- | --- | --- | --- | --- | --- | --- | --- | --- | --- | --- | --- | --- | --- | --- | --- | --- | --- | --- | --- | --- | --- | --- | --- | --- | --- | --- | --- | --- | --- | --- | --- | --- | --- | --- | --- | --- | --- | --- | --- | --- | --- | --- | --- | --- | --- | --- | --- | --- | --- | --- | --- | --- | --- | --- | --- | --- | --- | --- | --- | --- | --- | --- | --- | --- | --- | --- | --- | --- | --- | --- | --- | --- | --- | --- | --- | --- | --- | --- | --- | --- | --- | --- | --- | --- | --- | --- | --- | --- | --- | --- | --- | --- | --- | --- | --- | --- | --- | --- | --- | --- | --- | --- | --- | --- | --- | --- | --- | --- | --- | --- | --- | --- | --- | --- | --- | --- | --- | --- | --- | --- | --- | --- | --- | --- | --- | --- | --- | --- | --- | --- | --- | --- | --- | --- | --- | --- | --- | --- | --- | --- | --- | --- | --- | --- | --- | --- | --- | --- | --- | --- | --- | --- | --- | --- | --- | --- | --- | --- | --- | --- | --- | --- | --- | --- | --- | --- | --- | --- | --- | --- | --- | --- | --- | --- | --- | --- | --- | --- | --- | --- | --- | --- | --- | --- | --- | --- | --- | --- | --- | --- | --- | --- | --- | --- | --- | --- | --- | --- | --- | --- | --- | --- | --- | --- | --- | --- | --- | --- | --- | --- | --- | --- | --- | --- | --- | --- | --- | --- | --- | --- | --- | --- | --- | --- | --- | --- | --- | --- | --- | --- | --- | --- | --- | --- | --- | --- | --- | --- | --- | --- | --- | --- | --- | --- | --- | --- | --- | --- | --- | --- | --- | --- | --- | --- | --- | --- | --- | --- | --- | --- | --- | --- | --- | --- | --- | --- | --- | --- | --- | --- | --- | --- | --- | --- | --- | --- | --- | --- | --- | --- | --- | --- | --- | --- | --- | --- | --- | --- | --- | --- | --- | --- | --- | --- | --- | --- | --- | --- | --- | --- | --- | --- | --- | --- | --- | --- | --- | --- | --- | --- | --- | --- | --- | --- | --- | --- | --- | --- | --- | --- | --- | --- | --- | --- | --- | --- | --- | --- | --- | --- | --- | --- | --- | --- | --- | --- | --- | --- | --- | --- | --- | --- | --- | --- | --- | --- | --- | --- | --- | --- | --- | --- | --- | --- | --- | --- | --- | --- | --- | --- | --- | --- | --- | --- | --- | --- | --- | --- | --- | --- | --- | --- | --- | --- | --- | --- | --- | --- | --- | --- | --- | --- | --- | --- | --- | --- | --- | --- | --- | --- | --- | --- | --- | --- | --- | --- | --- | --- | --- | --- | --- | --- | --- | --- | --- | --- | --- | --- | --- | --- | --- | --- | --- | --- | --- | --- | --- | --- | --- | --- | --- | --- | --- | --- | --- | --- | --- | --- | --- | --- | --- | --- | --- | --- | --- | --- | --- | --- | --- | --- | --- | --- | --- | --- | --- | --- | --- | --- | --- | --- | --- | --- | --- | --- | --- | --- | --- | --- | --- | --- | --- | --- | --- | --- | --- | --- | --- | --- | --- | --- | --- | --- | --- | --- | --- | --- | --- | --- | --- | --- | --- | --- | --- | --- | --- | --- | --- | --- | --- | --- | --- | --- | --- | --- | --- | --- | --- | --- | --- | --- | --- | --- | --- | --- | --- | --- | --- | --- | --- | --- | --- | --- | --- | --- | --- | --- | --- | --- | --- | --- | --- | --- | --- | --- | --- | --- | --- | --- | --- | --- | --- | --- | --- | --- | --- | --- | --- | --- | --- | --- | --- | --- | --- | --- | --- | --- | --- | --- | --- | --- | --- | --- | --- | --- | --- | --- | --- | --- | --- | --- | --- | --- | --- | --- | --- | --- | --- | --- | --- | --- | --- | --- | --- | --- | --- | --- | --- | --- | --- | --- | --- | --- | --- | --- | --- | --- | --- | --- | --- | --- | --- | --- | --- | --- | --- | --- | --- | --- | --- | --- | --- | --- | --- | --- | --- | --- | --- | --- | --- | --- | --- | --- | --- | --- | --- | --- | --- | --- | --- | --- | --- | --- | --- | --- | --- | --- | --- | --- | --- | --- | --- | --- | --- | --- | --- | --- | --- | --- | --- | --- | --- | --- | --- | --- | --- | --- | --- | --- | --- | --- | --- | --- | --- | --- | --- | --- | --- | --- | --- | --- | --- | --- | --- | --- | --- | --- | --- | --- | --- | --- | --- | --- | --- | --- | --- | --- | --- | --- | --- | --- | --- | --- | --- | --- | --- | --- | --- | --- | --- | --- | --- | --- | --- | --- | --- | --- | --- | --- | --- | --- | --- | --- | --- | --- | --- | --- | --- | --- | --- | --- | --- | --- | --- | --- | --- | --- | --- | --- | --- | --- | --- | --- | --- | --- | --- | --- | --- | --- | --- | --- | --- | --- | --- | --- | --- | --- | --- | --- | --- | --- | --- | --- | --- | --- | --- | --- | --- | --- | --- | --- | --- | --- | --- | --- | --- | --- | --- | --- | --- | --- | --- | --- | --- | --- | --- | --- | --- | --- | --- | --- | --- | --- | --- | --- | --- | --- | --- | --- | --- | --- | --- | --- | --- | --- | --- | --- | --- | --- | --- | --- | --- | --- | --- | --- | --- | --- | --- | --- | --- | --- | --- | --- | --- | --- | --- | --- | --- | --- | --- | --- | --- | --- | --- | --- | --- | --- | --- | --- | --- | --- | --- | --- | --- | --- | --- | --- | --- | --- | --- | --- | --- | --- | --- | --- | --- | --- | --- | --- | --- | --- | --- | --- | --- | --- | --- | --- | --- | --- | --- | --- | --- | --- | --- | --- | --- | --- | --- | --- | --- | --- | --- | --- | --- | --- | --- | --- | --- | --- | --- | --- | --- | --- | --- | --- | --- | --- | --- | --- | --- | --- | --- | --- | --- | --- | --- | --- | --- | --- | --- | --- | --- | --- | --- | --- | --- | --- | --- | --- | --- | --- | --- | --- | --- | --- | --- | --- | --- | --- | --- | --- | --- | --- | --- | --- | --- | --- | --- | --- | --- | --- | --- | --- | --- | --- | --- | --- | --- | --- | --- | --- | --- | --- | --- | --- | --- | --- | --- | --- | --- | --- | --- | --- | --- | --- | --- | --- | --- | --- | --- | --- | --- | --- | --- | --- | --- | --- | --- | --- | --- | --- | --- | --- | --- | --- | --- | --- | --- | --- | --- | --- | --- | --- | --- | --- | --- | --- | --- | --- | --- | --- | --- | --- | --- | --- | --- | --- | --- | --- | --- | --- | --- | --- | --- | --- | --- | --- | --- | --- | --- | --- | --- | --- | --- | --- | --- | --- | --- | --- | --- | --- | --- | --- | --- | --- | --- | --- | --- | --- | --- | --- | --- | --- | --- | --- | --- | --- | --- | --- | --- | --- | --- | --- | --- | --- | --- | --- | --- | --- | --- | --- | --- | --- | --- | --- | --- | --- | --- | --- | --- | --- | --- | --- | --- | --- | --- | --- | --- | --- | --- | --- | --- | --- | --- | --- | --- | --- | --- | --- | --- | --- | --- | --- | --- | --- | --- | --- | --- | --- | --- | --- | --- | --- | --- | --- | --- | --- | --- | --- | --- | --- | --- | --- | --- | --- | --- | --- | --- | --- | --- | --- | --- | --- | --- | --- | --- | --- | --- | --- | --- | --- | --- | --- | --- | --- | --- | --- | --- | --- | --- | --- | --- | --- | --- | --- | --- | --- | --- | --- | --- | --- | --- | --- | --- | --- | --- | --- | --- | --- | --- | --- | --- | --- | --- | --- | --- | --- | --- | --- | --- | --- | --- | --- | --- | --- | --- | --- | --- | --- | --- | --- | --- | --- | --- | --- | --- | --- | --- | --- | --- | --- | --- | --- | --- | --- | --- | --- | --- | --- | --- | --- | --- | --- | --- | --- | --- | --- | --- | --- | --- | --- | --- | --- | --- | --- | --- | --- | --- | --- | --- | --- | --- | --- | --- | --- | --- | --- | --- | --- | --- | --- | --- | --- | --- | --- | --- | --- | --- | --- | --- | --- | --- | --- | --- | --- | --- | --- | --- | --- | --- | --- | --- | --- | --- | --- | --- | --- | --- | --- | --- | --- | --- | --- | --- | --- | --- | --- | --- | --- | --- | --- | --- | --- | --- | --- | --- | --- | --- | --- | --- | --- | --- | --- | --- | --- | --- | --- | --- | --- | --- | --- | --- | --- | --- | --- | --- | --- | --- | --- | --- | --- | --- | --- | --- | --- | --- | --- | --- | --- | --- | --- | --- | --- | --- | --- | --- | --- | --- | --- | --- | --- | --- | --- | --- | --- | --- | --- | --- | --- | --- | --- | --- | --- | --- | --- | --- | --- | --- | --- | --- | --- | --- | --- |

^a^QVAL: Phred scale q-value; ^b^RVSB: Relative Variant Strand Biais; ^c^AF : Allelic Fraction
