# Supplementary material for: KRAS mutations in blood circulating cell-free DNA: a pancreatic cancer case-control
Source: Oncotarget. 2016 Oct 1;7(48):78827–40. doi: 10.18632/oncotarget.12386 (PMC5346680; doi:10.18632/oncotarget.12386)
Supplement: Supplementary file 5 [file oncotarget-07-78827-s005.docx]

Supplementary Table S4: List of samples with cfDNA *KRAS* mutations outside of hotspot codons but reported in COSMIC

| \| Status \| Sample ID \| Stage \| \| QVAL^a^ \| \| Read Depth \| \| Mutated Reads \| \| RVSB^b^ \| \| HGVS cDNA \| \| HGVS protein \| \| Mutation type \| AF^c^ (%) \| COSMIC ID and occurrence \| \| --- \| --- \| --- \| --- \| --- \| --- \| --- \| --- \| --- \| --- \| --- \| --- \| --- \| --- \| --- \| --- \| --- \| --- \| --- \| \| PDAC \| CA86 \| Local \| 36.22 \| \| 2188 \| \| 8 \| \| 0.53 \| \| c.199A>C \| \| p.M67L \| \| missense \| \| 0.37 \| ID=COSM329471;OCCURENCE=1(haematopoietic_and_lymphoid_tissue) \| \| \| PDAC \| CA87 \| Regional \| 32.98 \| \| 2404 \| \| 9 \| \| 0.78 \| \| c.182A>C \| \| p.Q61P \| \| missense \| \| 0.37 \| ID=COSM551; OCCURRENCE=5(haematopoietic_and_lymphoid_tissue), 3(Thyroid), 2(Cervix), 2(Lung),1(Upper aerodigestive tract) \| \| \| Unknown/Missing \| CA85 \| Regional \| 242.26 \| \| 5310 \| \| 19 \| \| 0.58 \| \| c.190T>G \| \| p.Y64D \| \| missense \| \| 0.36 \| ID=COSM5044325; OCCURRENCE=1(haematopoietic_and_lymphoid_tissue) \| \| \| Endocrine \| CA88 \| Regional \| 241.03 \| \| 4047 \| \| 59 \| \| 0.50 \| \| c.179G>A \| \| p.G60D \| \| missense \| \| 1.46 \| ID=COSM87290;OCCURENCE=2(haematopoietic_and_lymphoid_tissue) \| \| \| Unknown/Missing \| CA82 \| Regional \| 70.36 \| \| 12324 \| \| 20 \| \| 0.63 \| \| c.176C>A \| \| p.A59E \| \| missense \| \| 0.16 \| ID=COSM1318029,COSM547;OCCURENCE=2(haematopoietic_and_lymphoid_tissue),1(endometrium),2(large_intestine) \| \| \| Unknown/Missing \| CA84 \| Regional \| 31.29 \| \| 2981 \| \| 7 \| \| 1.00 \| \| c.A209C \| \| p.Q70P \| \| missense \| \| 0.23 \| ID=COSM5008063, OCCURENCE=1(large_intestine) \| \| \| PDAC \| CA47 \| Systemic \| 671.66 \| \| 4529 \| \| 89 \| \| 0.69 \| \| c.180T>G \| \| p.G60G \| \| silent \| \| 1.97 \| ID=COSM1168050;OCCURENCE=2(large_intestine) \| \| \| PDAC \| CA89 \| Systemic \| 45.28 \| \| 2680 \| \| 44 \| \| 0.62 \| \| c.A214G \| \| p.M72V \| \| missense \| \| 1.64 \| ID=COSM1360840,COSM1360839;OCCURENCE=2(large_intestine) \| \| \| Endocrine \| CA83 \| Systemic \| 34.36 \| \| 6035 \| \| 13 \| \| 0.63 \| \| c.176C>G \| \| p.A59G \| \| missense \| \| 0.22 \| ID=COSM28518,COSM1135365;OCCURENCE=2(large_intestine),1(kidney) \| \| \| PDAC \| CA01 \| Systemic \| 41.23 \| \| 3301 \| \| 19 \| \| 0.67 \| \| c.28G>A \| \| p.G10R \| \| missense \| \| 0.58 \| ID=COSM1360887,COSM1360888;OCCURENCE=1(large_intestine) \| \| \| PDAC \| CA01 \| Systemic \| 34.05 \| \| 2746 \| \| 27 \| \| 0.54 \| \| c.14A>G \| \| p.K5R \| \| missense \| \| 0.98 \| ID=COSM1360894,COSM1360895;OCCURENCE=2(large_intestine) \| \| \| PDAC \| CA07 \| Systemic \| 44.01 \| \| 3048 \| \| 32 \| \| 0.51 \| \| c.14A>G \| \| p.K5R \| \| missense \| \| 1.05 \| ID=COSM1360894,COSM1360895;OCCURENCE=2(large_intestine) \| \| \| Unknown/Missing \| CA91 \| Unknown \| 35.60 \| \| 3461 \| \| 7 \| \| 0.50 \| \| c.186G>T \| \| p.E62D \| \| missense \| \| 0.20 \| ID=COSM556;OCCURENCE=1(endometrium) \| \| \| Benign pancreatic neoplasm \| BE2 \| NA \| 42.66 \| \| 3865 \| \| 5 \| \| 0.51 \| \| c.31G>C \| \| p.A11P \| \| missense \| \| 0.13 \| ID=COSM510;OCCURENCE=1(stomach),1(lung) \| \| \| Chronic pancreatitis \| CH03 \| NA \| 51.36 \| \| 5649 \| \| 11 \| \| 0.56 \| \| c.45C>A \| \| p.G15G \| \| silent \| \| 0.19 \| ID=COSM1360879,COSM1360878;OCCURENCE=1(large_intestine) \| \| \| Chronic pancreatitis \| CH04 \| NA \| 31.25 \| \| 3863 \| \| 19 \| \| 0.65 \| \| c.187G>A \| \| p.E63K \| \| missense \| \| 0.49 \| ID=COSM27159,COSM1360847;OCCURENCE=1(thyroid),1(central_nervous_system),1(genital_tract),3(large_intestine) \| \| \| Healthy control \| CO07 \| NA \| 36.52 \| \| 4794 \| \| 5 \| \| 0.63 \| \| c.181C>G \| \| p.Q61E \| \| missense \| \| 0.10 \| ID=COSM550; OCCURRENCE=5(lung), 3 (Central Nervous System), 2 (Large intestine), 1(urinary tract) \| \| \| Healthy control \| CO10 \| NA \| 38.35 \| \| 4141 \| \| 8 \| \| 0.70 \| \| c.186G>T \| \| p.E62D \| \| missense \| \| 0.19 \| ID=COSM556;OCCURENCE=1(endometrium) \| \| \| Healthy control \| CO08 \| NA \| 48.30 \| \| 7938 \| \| 12 \| \| 0.65 \| \| c.45C>A \| \| p.G15G \| \| silent \| \| 0.15 \| ID=COSM1360879,COSM1360878;OCCURENCE=1(large_intestine) \| \| \| Healthy control \| CO15 \| NA \| 426.17 \| \| 3148 \| \| 706 \| \| 0.53 \| \| c.175G>A \| \| p.A59T \| \| missense \| \| 22.43 \| ID=COSM546,COSM1562187;OCCURENCE=1(urinary_tract),7(large_intestine),3(salivary_gland),1(peritoneum),1(haematopoietic_and_lymphoid_tissue),5(stomach) \| \| \| Healthy control \| CO09 \| NA \| 30.08 \| \| 3027 \| \| 5 \| \| 1.00 \| \| c.45C>G \| \| p.G15G \| \| silent \| \| 0.17 \| ID=COSM1360879,COSM1360878;OCCURENCE=1(large_intestine) \| \| \| Healthy control \| CO11 \| NA \| 68.18 \| \| 4163 \| \| 9 \| \| 0.58 \| \| c.204G>C \| \| p.R68S \| \| missense \| \| 0.22 \| ID=COSM98455;OCCURENCE=1(large_intestine) \| \| |
| --- | --- | --- | --- | --- | --- | --- | --- | --- | --- | --- | --- | --- | --- | --- | --- | --- | --- | --- | --- | --- | --- | --- | --- | --- | --- | --- | --- | --- | --- | --- | --- | --- | --- | --- | --- | --- | --- | --- | --- | --- | --- | --- | --- | --- | --- | --- | --- | --- | --- | --- | --- | --- | --- | --- | --- | --- | --- | --- | --- | --- | --- | --- | --- | --- | --- | --- | --- | --- | --- | --- | --- | --- | --- | --- | --- | --- | --- | --- | --- | --- | --- | --- | --- | --- | --- | --- | --- | --- | --- | --- | --- | --- | --- | --- | --- | --- | --- | --- | --- | --- | --- | --- | --- | --- | --- | --- | --- | --- | --- | --- | --- | --- | --- | --- | --- | --- | --- | --- | --- | --- | --- | --- | --- | --- | --- | --- | --- | --- | --- | --- | --- | --- | --- | --- | --- | --- | --- | --- | --- | --- | --- | --- | --- | --- | --- | --- | --- | --- | --- | --- | --- | --- | --- | --- | --- | --- | --- | --- | --- | --- | --- | --- | --- | --- | --- | --- | --- | --- | --- | --- | --- | --- | --- | --- | --- | --- | --- | --- | --- | --- | --- | --- | --- | --- | --- | --- | --- | --- | --- | --- | --- | --- | --- | --- | --- | --- | --- | --- | --- | --- | --- | --- | --- | --- | --- | --- | --- | --- | --- | --- | --- | --- | --- | --- | --- | --- | --- | --- | --- | --- | --- | --- | --- | --- | --- | --- | --- | --- | --- | --- | --- | --- | --- | --- | --- | --- | --- | --- | --- | --- | --- | --- | --- | --- | --- | --- | --- | --- | --- | --- | --- | --- | --- | --- | --- | --- | --- | --- | --- | --- | --- | --- | --- | --- | --- | --- | --- | --- | --- | --- | --- | --- | --- | --- | --- | --- | --- | --- | --- | --- | --- | --- | --- | --- | --- | --- | --- | --- | --- | --- | --- | --- | --- | --- | --- | --- | --- | --- | --- | --- | --- | --- | --- | --- | --- | --- | --- | --- | --- | --- | --- | --- | --- | --- | --- | --- | --- | --- | --- | --- | --- | --- | --- | --- | --- | --- | --- | --- | --- | --- | --- | --- | --- | --- | --- | --- | --- | --- | --- | --- | --- | --- | --- | --- | --- | --- | --- | --- | --- | --- | --- | --- | --- | --- | --- | --- | --- | --- | --- | --- | --- | --- | --- | --- | --- | --- | --- | --- | --- | --- | --- | --- | --- | --- | --- | --- | --- | --- | --- | --- | --- | --- | --- | --- | --- | --- | --- | --- | --- | --- | --- | --- | --- | --- | --- | --- | --- | --- | --- | --- | --- | --- | --- | --- | --- | --- | --- | --- | --- | --- | --- | --- | --- | --- | --- | --- | --- | --- | --- | --- | --- | --- | --- | --- | --- | --- | --- | --- | --- | --- | --- | --- | --- | --- | --- | --- | --- | --- | --- | --- | --- | --- | --- | --- | --- | --- | --- | --- | --- | --- | --- | --- | --- | --- | --- | --- | --- | --- | --- |

^a^QVAL: Phred scale q-value; ^b^RVSB: Relative Variant Strand Biais; ^c^AF : Allelic Fraction

**Data and materials availability**: We provide the URL link for the free use of the Needlestack algorithm (https://github.com/IARCbioinfo/needlestack)
